# Structural and biochemical investigation into stable FGF2 mutants with novel mutation sites and hydrophobic replacements for surface-exposed cysteines

**DOI:** 10.1371/journal.pone.0307499

**Published:** 2024-09-05

**Authors:** Young Jun An, Ye-Eun Jung, Kyeong Won Lee, Prashant Kaushal, In Young Ko, Seung Min Shin, Sangho Ji, Wookyung Yu, Cheolju Lee, Won-Kyu Lee, Kiweon Cha, Jung-Hyun Lee, Sun-Shin Cha, Hyung-Soon Yim

**Affiliations:** 1 Marine Biotechnology & Bioresource Research Department, Korea Institute of Ocean Science and Technology, Busan, Republic of Korea; 2 Department of Chemistry and Nanoscience, Ewha Womans University, Seoul, Republic of Korea; 3 Chemical & Biological Integrative Research Center, Korea Institute of Science and Technology, Seoul, Republic Korea; 4 New Drug Development Center, Osong Medical Innovation Foundation, Cheongiu, Republic of Korea; 5 Department of Brain Sciences, DGIST, Daegu, Republic of Korea; Université Sorbonne Paris Nord: Universite Sorbonne Paris Nord, FRANCE

## Abstract

Fibroblast growth factor 2 (FGF2) is an attractive biomaterial for pharmaceuticals and functional cosmetics. To improve the thermo-stability of FGF2, we designed two mutants harboring four-point mutations: FGF2-M1 (D28E/C78L/C96I/S137P) and FGF2-M2 (D28E/C78I/C96I/S137P) through bioinformatics, molecular thermodynamics, and molecular modeling. The D28E mutation reduced fragmentation of the FGF2 wild type during preparation, and the substitution of a whale-specific amino acid, S137P, enhanced the thermal stability of FGF2. Surface-exposed cysteines that participate in oligomerization through intermolecular disulfide bond formation were substituted with hydrophobic residues (C78L/C78I and C96I) using the *in silico* method. High-resolution crystal structures revealed at the atomic level that the introduction of mutations stabilizes each local region by forming more favorable interactions with neighboring residues. In particular, P137 forms CH-π interactions with the side chain indole ring of W123, which seems to stabilize a β-hairpin structure, containing a heparin-binding site of FGF2. Compared to the wild type, both FGF2-M1 and FGF2-M2 maintained greater solubility after a week at 45 °C, with their *T*_m_ values rising by ~ 5 °C. Furthermore, the duration for FGF2-M1 and FGF2-M2 to reach 50% residual activity at 45 °C extended to 8.8- and 8.2-fold longer, respectively, than that of the wild type. Interestingly, the hydrophobic substitution of surface-exposed cysteine in both FGF2 mutants makes them more resistant to proteolytic cleavage by trypsin, subtilisin, proteinase K, and actinase than the wild type and the Cys → Ser substitution. The hydrophobic replacements can influence protease resistance as well as oligomerization and thermal stability. It is notable that hydrophobic substitutions of surface-exposed cysteines, as well as D28E and S137P of the FGF2 mutants, were designed through various approaches with structural implications. Therefore, the engineering strategies and structural insights adopted in this study could be applied to improve the stability of other proteins.

## Introduction

The human fibroblast growth factor (FGF) family consists of 18 members (FGF1–FGF10 and FGF16–FGF23) [1, 2] known to be involved in morphogen and growth factor functions in development and homeostasis [2–5]. Canonical FGFs including FGF2 act as a paracrine and/or an autocrine by forming ternary complexes with FGF receptors (FGFRs) and heparan sulfate proteoglycans (HSPGs) on the cell surface [6–8]. The FGFR-FGF-HSPG complex induces the dimerization of FGFR, resulting in a series of intracellular signaling cascades including the activation of MAPK/ERK pathway, PI3K/AKT pathway, and PLCγ pathway, etc [9, 10]. Through these signaling pathways, FGF2 regulates diverse cellular responses and has beneficial effects on wound healing, wrinkle reduction, and protection against neomycin-induced hair cell loss [11, 12]. Therefore, it has attracted attention in the pharmaceutical and cosmetic fields.

While the therapeutic potentials of FGF2 are widely recognized, a significant challenge hindering its broader application is inherent instability [12, 13]. Furthermore, thermo-stability and protease resistance are required to prevent protein denaturation during production and long-term storage. Previous studies have reported some strategies to improve the stability of FGF through genetic modification [14–17], structural analysis [18–20], and computational approaches [12, 21, 22], etc. While these strategies are promising, most FGF2 variants possess limitations that necessitate further optimization to enhance the potential of FGF2 [15]. Consequently, it remains important to explore various approaches, such as atomic-level structural analysis, to improve the stability of FGF2 and provide insights for its enhancement. This study aimed to identify novel mutation sites in FGF2 and apply a hydrophobic replacement strategy targeting surface-exposed cysteines to enhance protein stability.

Protein engineering has been employed by using genetic information to enhance protein stability [23, 24]. Especially, the distinctive traits of marine life have a lot of industrial potential, leading to more research on utilizing the genetic information from marine organisms [25]. Calcitonin, a peptide hormone composed of 32 amino acids, has the ability to regulate blood calcium levels [26] and is effective in treating Paget’s disease of bone, a chronic disease of the skeleton [27]. Notably, salmon calcitonin is used as an alternative to human calcitonin [28] because of its high stability and efficacy against Paget’s disease [29]. In addition, the evolutionary analysis of cetacean FGFs has provided insight into the adaptation of cetaceans to aquatic environments [30]. The cetacean genome-based design of the FGF21 variant successfully enhanced its potency for FGFR1c/β-Klotho activation than the wild type [31]. Interestingly, whale exhibits great wound-healing capacity [32, 33]. Regarding FGF2’s role in wound healing, it is plausible that whale FGF2 could contribute to the regenerative ability of the skin. Therefore, we chose the whale-specific sequence (S137P) as a novel mutation site to improve its stability and efficacy. Besides, the D28E mutation was introduced into human FGF2 through mass analysis of fragmented FGF2 to protect against the proteolytic sites.

Intramolecular disulfide bonds between cysteine residues contribute to protein stability [34]. Conversely, cysteines exposed on the surface can cause aggregation by forming intermolecular disulfide bonds [35]. In previous studies, it has been shown that two surface-exposed cysteines (C78 and C96) of FGF2 play a crucial role in extracellular secretion via oligomerization [36, 37]. On the other hand, these cysteines were targeted for the major mutation sites, being replaced by alanine [37], serine, or asparagine to prevent aggregation in the production of recombinant FGF2 [12, 38]. In this study, we implemented a hydrophobic replacement approach for the surface-exposed cysteines by using *in silico* energy calculations. Additionally, we solved the high-resolution crystal structures of the FGF2 mutants harboring both the novel mutation sites and the hydrophobic replacements to elucidate their stabilizing effects. These approaches showed better enhancement of stability for FGF2, suggesting the structural insights on modifying the structural and physicochemical characteristics of proteins to enhance their therapeutic potential.

## Materials and methods

### Cloning, expression, and purification of FGF2

The genes for human (*Homo sapiens)* proFGF2 (residues 1–155) and Δ9N_FGF2 (residues 10–155) were synthesized at Bionics Co., Ltd. (Republic of Korea). They were inserted at the *Sal*I and *Hind*III sites of the expression vector pQE80 (Qiagen, Germany), and the *Nde*I and *Xho*I sites of the expression vector pET17b(+) (Novagen, USA), respectively (S1 Fig). We initially used the pQE80 vector for expressing the FGF2 protein. This vector includes a His tag at the N-terminal of FGF2 without a cleavage site. To produce the native FGF2, we utilized the pET17b vector. proFGF2 contains residues 1–9 and Δ9N_FGF2 does not. The construct was transformed into *Escherichia coli* strain Rosetta (DE3) pLysS (Stratagene, La Jolla, CA, USA).

The transformed cells were grown at 37 °C in a Luria-Bertani medium containing 50 μg/mL to an OD_600_ of ~ 0.6. Afterward, FGF2 expression was induced by adding 0.5 mM isopropyl β-D-thiogalactopyranoside. After an 18 h induction at 20 °C, cells were harvested and resuspended in a buffer containing 20 mM Tris (pH 8.0) and 100 mM NaClfor sonication. The cell debris was discarded by centrifugation at 13,000 rpm for 40 min at 4 °C. The supernatants were loaded onto a heparin column (GE Healthcare, USA). The column was washed using buffers containing 20 mM Tris (pH 8.0) with 100 mM NaCl and 20 mM Tris (pH 8.0) with 500 mM NaCl. The elution of bound FGF2 was performed using 20 mM Tris buffer (pH 8.0) with a gradient of NaCl (500–1800 mM). The eluted fractions containing FGF2 were applied to a Superdex 75 HR 16/60 column (GE Healthcare, USA) equilibrated with 1X phosphate-buffered solution (PBS) buffer.

### Site-directed mutagenesis

The human *fgf2* gene was synthesized at Bionics Co., Ltd. Various mutants (proFGF2 D15E, proFGF2 D28E, proFGF2 T121S, proFGF2 S137P, proFGF2 T121S/S137P, FGF2 D28E, FGF2 S137P, FGF2 D28E/S137P, FGF2 C78L/C96I, FGF2 C78I/C96I, FGF2 C78A/C96A, FGF2 C78S/C96S, FGF2 D28E/C78L/C96I/S137P (FGF2-M1), and FGF2 D28E/C78I/C96I/S137P (FGF2-M2)) were obtained by site-directed mutagenesis. The details of the mutant proteins used in this study are shown in Table 1 and S1 Fig.

**Table 1:** Indication of the FGF2 name described in the manuscript.

Vector	Variant	Abbreviation
pQE80 (Residues 1–9 included)	proFGF2	pFGF2
	proFGF2 D15E	pFGF2 D15E
	proFGF2 D28E	pFGF2 D28E
	proFGF2 T121S	pFGF2 T121S
	proFGF2 S137P	pFGF2 S137P
	proFGF2 T121S/S137P	pFGF2 T121S/S137P
pET17b	Δ9N_FGF2 (Wild)	FGF2
	Δ9N_FGF2 D28E	FGF2 D28E
	Δ9N_FGF2 S137P	FGF2 S137P
	Δ9N_FGF2 D28E/S137P	FGF2 D28E/S137P
	Δ9N_FGF2 C78L/C96I	FGF2 C78L/C96I
	Δ9N_FGF2 C78I/C96I	FGF2 C78I/C96I
	Δ9N_FGF2 C78A/C96A	FGF2 C78A/C96A
	Δ9N_FGF2 C78S/C96S	FGF2 C78S/C96S
	Δ9N_FGF2 D28E/C78L/C96I/S137P	FGF2-M1
	Δ9N_FGF2 D28E/C78I/C96I/S137P	FGF2-M2

### Analysis of protein N-terminus by mass spectrometry

The N-terminal peptide FGF2 or its fragments was recovered from the corresponding protein band of sodium dodecylsulfate polyacrylamide gel electrophoresis (SDS-PAGE) by the GelNrich method as described in the paper [39]. Briefly, the protein band was cut and treated with 10 mM dithiothreitol (56 °C, 45 min) and 55 mM iodoacetamide (RT, 30 min, in the dark) in 50 mM EPPS buffer (pH 8). Next, the gel piece was dehydrated using acetonitrile, treated with 50 mM d6-acetic anhydride (Sigma Aldrich) in 0.2 M EPPS buffer (pH 8) at RT for 20 min to acetylate amino groups at the protein N-terminus and lysine residue side chains, and washed with the same buffer. This whole sequence of dehydration, N-acetylation, and washing was repeated three times. Next, the protein in the gel piece was digested with trypsin by the so-called in-gel digestion method and the peptides were redissolved in a buffer containing 0.2 M EPPS and 0.15 M NaCl (pH 8). In the mixture of tryptic peptides, internal peptides were depleted by using NHS-activated agarose beads. The resultant supernatant (comprising mostly the undepleted N-terminal peptide) was analyzed by nano-flow reversed-phase liquid chromatography-tandem mass spectrometry. Whether a peptide measured in mass spectrometry is N-terminal is determined by the acetylation status of the peptide. The N-terminal peptide appears as d3-acetylated or acetylated, while the internal peptide appears as unacetylated.

### Sequence analysis

The amino acid of FGF2 is a low molecular weight isoform (LMW) consisting of 155 amino acids and is approximately 18kDa. The FGF2 protein sequences of humans and four whale species (bowhead whale, sperm whale, fin whale, and minke whale) were compared and analyzed using the MultAlin [40] and ESPript [41] 3.0 servers.

### Energy calculation of the substitutions of the cysteine residues on the surface of FGF2

For mutation optimization of FGF2, the stability of mutations was assessed; the energy was calculated using two stability prediction tools: Site Directed Mutator (SDM) [42] and Discovery Studio (version 2021, Biovia, USA). The crystal structure of FGF2 mutants (C78S, C96S) (PDB ID:1ii4) molecular has been replaced by the wild type with the SWISS-MODEL server [43] for use as stability calculation models.

### Crystallization, data collection, and structure determination

FGF2-M1 and FG2-M2 in a buffer consisting of 20 mM Tris (pH 8.0) and 200 mM NaCl were concentrated to ~15 mg/mL and crystallized using the modified vapor batch crystallization method reported in our previous studies [44, 45]. Crystals of FGF2-M1 were grown in mother liquor consisting of 1.5 M sodium citrate and 100 mM sodium cacodylate (pH 5.5). To create a complex between FGF2-M2 and sucrose octa-sulfate (SOS), the proteins were mixed with 2 mM SOS. Crystals of the complex were grown in a mother liquor of 30% (w/v) polyethylene glycol 2K and 100 mM potassium thiocyanate.

For data collection, crystals were transferred into the mother liquors supplemented with 20% glycerol as a cryoprotectant. The samples were mounted in a stream of liquid nitrogen at 100 K. The X-ray diffraction data of FGF2-M1 and the FGF2-M2/SOS complex were collected to the resolution of 1.40 Å and 1.48 Å, respectively, at the beamline 11C of Pohang Light Source installed by a Pilatus3 6M (Dectris) detector (Pohang Light Source, Republic of Korea) (S1 Table). The collected data were processed using the DENZO and SCALEPACK programs available in the HKL2000 program suite (HKL Research, Inc., VA, USA) [46].

FGF2-M1 crystals belonged to the space group, *P*3_1_21 (a = b = 54.375 Å and c = 97.012 Å), with one molecule in the asymmetric unit (S1 Table). Molecular replacement with the available FGF2 structure (PDB code: 4OEE) as a search model was successful using Phaser-MR [47] in the PHENIX program suit [48]. The capital and small letters of each program should be consistent. Several rounds of manual refitting and refinement were performed with Coot [49] and Phenix [48], respectively. The final model of FGF-M1 with *R*_work_/*R*_free_ of 18.51/21.27% contains residues 20–154 and 138 water molecules (S1 Table). Crystals of the FGF2-M2/SOS complex with three molecules in the unit cell belonged to the space group, *P*2_1_ (a = 51.38 Å, b = 54.45 Å, and c = 71.97 Å) (S1 Table). Molecular replacement, manual refitting, and refinement were performed using the same method described above. The final model of the FGF2-M2/SOS complex structure with *R*_work_/*R*_free_ = 17.91/19.56% contains residues 20–154, two SOS molecules, and 360 water molecules (S1 Table). *B*-factor values of the atoms were normalized by the following equation: *B*´ = (*B*—<*B*>)/σ(*B*), in which <*B*> and σ(*B*) represented the average value of *B*-factors and the standard deviation of the *B*-factor values, respectively [50].

### Thermal stability of FGF2 proteins assessed using sodium dodecyl-sulfate polyacrylamide gel electrophoresis

Protein samples (0.5 mg/mL, volume 40 μL) were mixed with 1X PBS (137 mM NaCl, 2.7 mM KCl, 10 mM Na_2_HPO_4_, 1.8 mM KH_2_PO_4_ at a pH of 7.4). The proFGF2 mutants were incubated at 37 °C for 6 days and sampled at an interval of two days. FGF2 mutants with surface cysteine substitution were incubated for seven days. Soluble proteins were collected by centrifugation (12,000 rpm for 10 min) and analyzed using 15% SDS-PAGE. The relative amount of soluble protein as a percentage of each protein band was quantified using the ImageJ software.

### Reversed-phase HPLC analysis

10 μL of 1 mg/mL protein was injected onto an Xbridge Protein BEH300 C4 column 300 Å, 3.5 μm, 4.6 × 150 mm; Waters) using solution A (0.1% FA in water) and solution B (0.1% FA in acetonitrile). The sample was separated with a gradient (20–50% of B) for 20 min at a column temperature of 80 °C and a flow rate of 1.44 mL/min. Peak areas were integrated using Agilent Chemstation software (Agilent, USA). All samples were thawed and centrifuged at 10,000 rpm for 10 minutes to remove aggregation, and only the supernatant was analyzed.

### Circular dichroism (CD) spectroscopy

The CD measurement was performed using a Jasco J-1500 with a PTC-517 Peltier thermostatted cell holder (Jasco Corporation, Japan) at the National Research Facilities and Equipment Center (NanoBio-Energy Materials Center) at Ewha Womans University. FGF2 concentration was ~0.2 mg/mL in a quartz cuvette (path length of 1 mm; Jasco Corporation). Thermal denaturation measurements were performed at 228 nm with a heating rate of 1 °C/min and an interval of 1 °C from 20 to 100 °C. Data were collected by continuous scanning at a CD scale of 200 mdeg/0.1 dOD, a digital integration time of 2 s, and a bandwidth of 1.00 nm. The values of the protein-free PBS buffer were subtracted from each signal in the CD spectrum. The transition curve representing the denaturation state of the protein sample in the 0–100% range was developed using the standard equation: % denaturation = ((θ_20_−θ_temp_)/(θ_20_−θ_100_)) × 100; θ_temp_ indicates the mean residue molar ellipticity of the protein at a specific temperature. The curves were fitted using a nonlinear adjustment by the Boltzmann method of the OriginPro 2021 program suite (Origin Lab Corporation, USA). The inflection point of the curve corresponds to *T*_m_. Each average *T*_m_ value with a standard deviation was calculated from three independent experiments (S3 Table).

### Protease resistance assay

Proteolysis was conducted using Proti-Ace (α-chymotrypsin, trypsin, elastase, papain, subtilisin, and endoproteinase Glu-C) and Proti-Ace 2 (proteinase K, clostripain, pepsin, thermolysin, bromelain, and actinase), which consist of 12 proteases from Hampton Research. Small-scale screening experiments were conducted on 30 μl samples of FGF2s at a concentration of 0.3 mg/ml. The protein and protease were mixed at a ratio of 1:100 for the experiment, and 1X PBS buffer (pH 7.4, 137 mM NaCl, 2.7 mM KCl, 10 mM Na_2_HPO_4_, 1.8 mM KH_2_PO_4_) was used. Protein degradation took place at 37 °C for 3 hours. A 10 μl sample was subjected to SDS-PAGE electrophoresis, and quantification was performed using the ImageJ program.

### Cell culture and cell proliferation assay

BALB3T3 cells were maintained in Dulbecco’s modified Eagle’s medium (DMEM; GIBCO, Life Technologies Ltd., Paisley, UK) supplemented with 10% bovine serum (BS; GIBCO) and 1% penicillin/streptomycin (GIBCO) at 37 °C in a 5% CO_2_ incubator. To assess cell proliferation activity, cells (0.365 × 10^4^/well) were seeded in 96-well plates and incubated with FGF2s in DMEM/F12 medium (GIBCO) containing 1 μM dexamethasone, 10 μg/mL insulin, 10 ng/mL sodium selenite, 10 μg/mL transferrin, 5 μg/mL fibronectin, 100 μg/mL ovalbumin, and 10 μg/mL heparin. After 40 h, the levels of proliferation were determined using the Cell Counting Kit-8 (CCK8; Dojindo, Gaithersburg, MD, USA), according to the manufacturer’s protocol. Results are expressed as the average ± standard error of the mean (SEM) of at least three independent experiments. Statistical significance was calculated using one-way analysis of variance (ANOVA) followed by Tukey’s post hoc test. A *P*-value of < 0.05 was considered statistically significant.

## Results and discussion

### Identification of a novel mutation site through sequence comparison between human and whale FGF2 sequences

**Fig 1:**
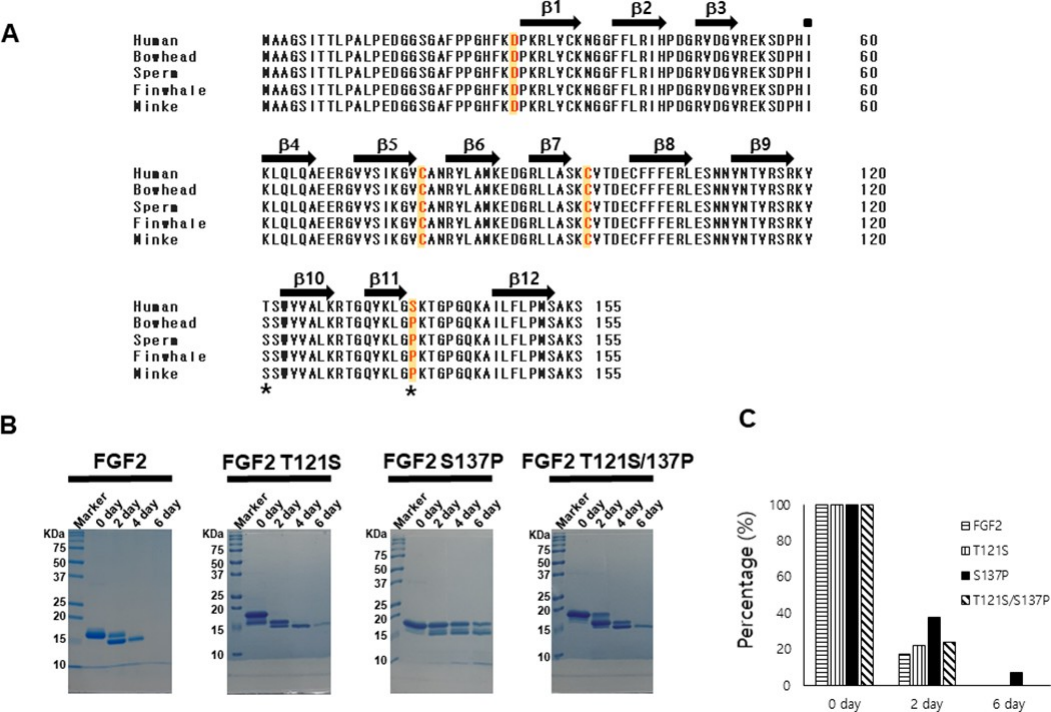
A comparative analysis of sequence and thermal stability in human and whale FGF2. (A) Mutation positions are indicated with yellow boxes and red text. The different sequences of humans and whales are indicated with a black star. Beta-strands are indicated by a black arrow. Protein sequence alignment was calculated using ESPript 3.0 (Robert and Gouet 2014) and MultAlin (Corpet 1988) servers. (B) The FGF2 mutants (T121S, S137P, T121S/S137P) with whale sequences are tested for thermal stability for six days at 37 °C, compared with wild-type human FGF2. Protein stability is demonstrated by SDS-PAGE gel and (C) Reverse phase HPLC. FGF2 variants (FGF2, FGF2 T121S, FGF2 S137P, FGF2 T121S/S137P) were tested for 6 days. The main protein bands of the FGF2 variants (FGF2 (▤), FGF2 T121S (▥), FGF2 S137P (■), and FGF2 T121S/S137P (▧) are calculated as percentages and shown as bar graphs.

A single FGF2 transcript contains five FGF2 isoforms. It can be translated into an LMW (18 kDa) isoform and four high molecular weight (HMW) isoforms (22, 22.5, 24, and 34 kDa) [51, 52]. In this study, we attempted to improve the stability of FGF2 by introducing whale-specific amino acids using LMW FGF2. Alignment of the sequences of FGF2s from human and four whale species (Bowhead, Sperm, Fin, and Minke) revealed 98.7% amino acid sequence identity. They differed by only two amino acids (T121S, S137P; Fig 1A). To examine the effect of the sequence disparities on the thermo-stability of FGF2, purified proFGF2 mutants with whale FGF2 sequences (T121S, S137P, and T121S/S137P) were incubated at 37 °C for 6 days and compared using SDS-PAGE gels. proFGF2s appeared to degrade into small-sized proteins, of which S137P retained its original size compared to proFGF2, as well as the whale FGF2 sequence-introduced T121S and T121S/S137P mutants (Fig 1B). Likewise, in the reversed-phase HPLC experiments, a major peak was observed only for the FGF2 S137P mutant among the FGF2 mutants on day 6 (Fig 1C and S2 Table). Based on these results, S137P from the whale FGF2 sequence was selected as the candidate for a mutation to improve protein stability.

### Discovery of a novel mutation site to prevent site-specific fragmentation of FGF2

**Fig 2:**
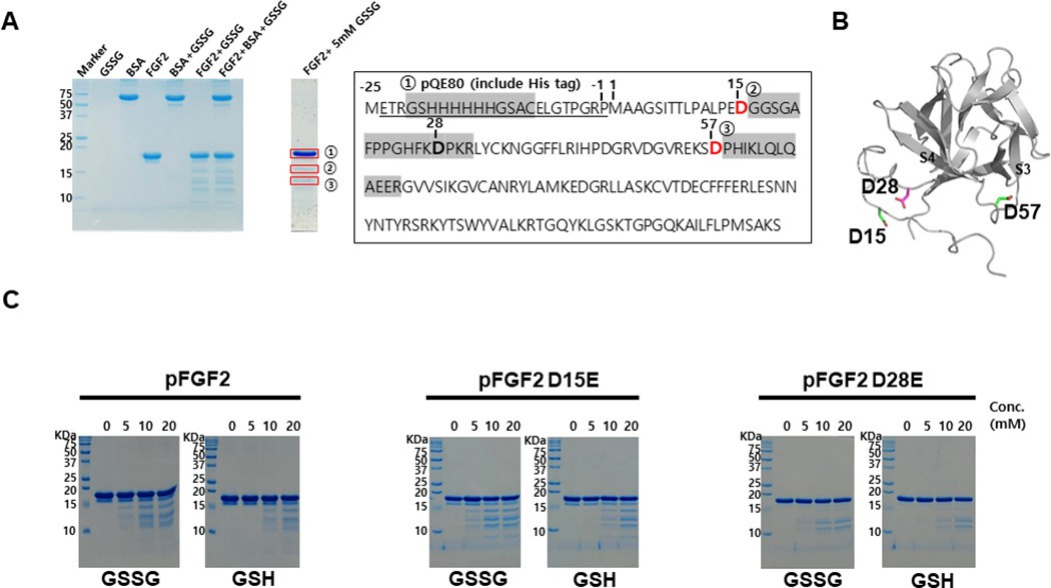
Glutathione effect of FGF2 mutants. (A) The result of 15% SDS-PAGE after incubating pFGF2 with 5 mM glutathione at 45 °C for 6 days. The bands analyzed by GelNrich-coupled mass spectrometry are marked in red boxes and labeled as ①, ②, and ③. The pFGF2 sequence is shown in the right box, with D15 and D57 indicated in red text. The major fragments of pFGF2 analyzed by N-terminal sequencing are highlighted in gray and labeled as ①, ②, and ③. (B) The structure of FGF2 with D15, D57 (green sticks), and D28 (a magenta stick). (C) The result of 15% SDS-PAGE after incubating pFGF2, pFGF2 D15E, and pFGF2 D28E with 0 ~ 20 mM GSSG and GSH, respectively.

S-glutathionylation is a reversible disulfide combination of cysteinyl thiol in the protein and thiol of glutathione [53]. Glutathionylation can protect against peroxidation or decomposition of proteins [54]. Cysteine on the surface of FGF2 is known to form oligomers between FGF2s and translocate FGF2 to the cell surface [37]. However, this feature causes protein aggregation during purification and concentration. Therefore, glutathione was mixed with FGF2 to prevent oligomerization. proFGF2 was incubated with 5 mM GSSG for six days in 1X PBS buffer and monitored by SDS-PAGE (Fig 2). The incubation of proFGF2 with GSSG yielded a specific fragmentation pattern of proFGF2. The mass analysis of three thick protein bands excised from SDS-PAGE gel identified distinct cleavage sites located before M1, after D15 (between Asp and Gly amino acids), and after D57 (between Asp and Pro amino acids) (Fig 2A, Left graph and sequence in S2 Fig). Based on observations (Fig 2 and S2 Fig), it seemed to be possible that the cleavage of FGF2 might occur due to contamination by a D selective protease. To prevent the site-specific fragmentation, proFGF2 mutants were constructed by considering the protein tertiary structure and sequences. In the FGF2 structure, D57, located between β3 and β4 strands (Figs 1A and 2B), was excluded from the mutation site (Fig 2) because the mutation can distort the structure of FGF2. There is one more aspartic acid D28 located between D15 and D57 at the starting point of the β-trefoil fold domain of FGF2. D28 can be another cleavage site even though this residue was not detected by mass analysis. The D15 and D28 residues in the N-terminal loop were replaced with glutamic acid (E). To verify the stability of proFGF2 mutations, the GSH and GSSG were incubated with two proFGF2 mutants (D15E and D28E) for 3 h at 37 °C to compare the fragmentation of proFGF2 mutant protein. proFGF2 D15E showed a similar fragmentation pattern to proFGF2, but proFGF2 D28E decreased protein fragmentation (Fig 2C). It has been shown that the introduction of D28E could reduce the FGF2 fragmentation by a D selective protease. Therefore, the D28E mutation was added to proFGF2 S137P to improve further stability of FGF2.

### A hydrophobic replacement strategy for surface-exposed cysteines by using *in silico* prediction

SDM and Discovery Studio 2019 programs were used to identify alternative amino acids that can prevent oligomerization and improve energy stability. To predict the stabilization of a region containing cysteine residues on the FGF2 surface, we replaced that region with 19 amino acids and calculated the energies of each. Calculations using the SDM program showed that C78 and C96 were most energy-stabilized when replaced by leucine (predicted pseudo ΔΔG: 0.33) and isoleucine (predicted pseudo ΔΔG: 0.19), respectively (Table 2). Discovery Studio revealed that C78 was most energy-stabilized when it was replaced by isoleucine (mutation energy: −1.2 kcal/mol). However, the energy stability of C96 did not differ significantly before and after being replaced by tryptophan (mutation energy: 0.39 kcal/mol) (Table 3). Based on the calculation by *in silico* predictions, two FGF2 mutants with four-point mutations (FGF2-M1 with D28E/C78L/C96I/S137P and FGF2-M2 with D28E/C78I/C96I/S137P) were finally designed.

**Table 2:** Prediction of FGF2 stability using SDM.

Wild type	Mutation	Predicted pseudo ΔΔG:	Stability
**C78**	**E**	**-0.05**	**Reduced**
**C78**	**S**	**-1.15**	**Reduced**
**C78**	**G**	**-0.46**	**Reduced**
**C78**	**A**	**0.09**	**Increased**
**C78**	**H**	**0.27**	**Increased**
**C78**	**D**	**-0.7**	**Reduced**
**C78**	**N**	**-0.48**	**Reduced**
**C78**	**Y**	**0.07**	**Increased**
**C78**	**F**	**0.27**	**Increased**
**C78**	**W**	**-0.01**	**Reduced**
**C78**	**V**	**-0.07**	**Reduced**
**C78**	**T**	**-0.69**	**Reduced**
**C78**	**L**	**0.33**	**Increased**
**C78**	**P**	**-0.71**	**Reduced**
**C78**	**R**	**0.12**	**Increased**
**C78**	**K**	**0.07**	**Increased**
**C78**	**Q**	**0.07**	**Increased**
**C78**	**M**	**-0.03**	**Reduced**
**C78**	**I**	**-0.07**	**Reduced**
**C96**	**E**	**-0.34**	**Reduced**
**C96**	**S**	**-0.82**	**Reduced**
**C96**	**G**	**-0.29**	**Reduced**
**C96**	**A**	**-0.76**	**Reduced**
**C96**	**H**	**0.09**	**Increased**
**C96**	**D**	**-0.28**	**Reduced**
**C96**	**N**	**-0.76**	**Reduced**
**C96**	**Y**	**0.09**	**Increased**
**C96**	**F**	**-0.2**	**Reduced**
**C96**	**W**	**0.02**	**Increased**
**C96**	**V**	**0.04**	**Increased**
**C96**	**T**	**-0.8**	**Reduced**
**C96**	**L**	**-0.2**	**Reduced**
**C96**	**P**	**-0.48**	**Reduced**
**C96**	**R**	**0.08**	**Increased**
**C96**	**K**	**-0.29**	**Reduced**
**C96**	**Q**	**-0.28**	**Reduced**
**C96**	**M**	**-0.08**	**Reduced**
**C96**	**I**	**0.19**	**Increased**

**Table 3:** Prediction of FGF2 stability using Discovery studio 2019.

**Index**	**Mutation**	**Mutation Energy(kcal/mol)**	**Effect**
**1**	**A: CYS78>ILE**	**-1.2**	**STABILIZING**
**2**	**A: CYS78>TRP**	**-0.99**	**STABILIZING**
**3**	**A: CYS78>TYR**	**-0.75**	**STABILIZING**
**4**	**A: CYS78>ASN**	**-0.66**	**STABILIZING**
**5**	**A: CYS78>ASP**	**-0.49**	**NEUTRAL**
**16**	**A: CYS78>HIS**	**0.67**	**DESTABILIZING**
**17**	**A: CYS78>ARG**	**0.92**	**DESTABILIZING**
**18**	**A: CYS78>GLY**	**1.49**	**DESTABILIZING**
**19**	**A: CYS78>LYS**	**1.96**	**DESTABILIZING**
**20**	**A: CYS78>PRO**	**2.3**	**DESTABILIZING**
**Index**	**Mutation**	**Mutation Energy(kcal/mol)**	**Effect**
**1**	**A: CYS96>TRP**	**-0.39**	**NEUTRAL**
**2**	**A: CYS96>CYS**	**-0.38**	**NEUTRAL**
**3**	**A: CYS96>ASP**	**-0.19**	**NEUTRAL**
**4**	**A: CYS96>ILE**	**0.03**	**NEUTRAL**
**5**	**A: CYS96>ASN**	**0.17**	**NEUTRAL**
**16**	**A: CYS96>TYR**	**1.14**	**DESTABILIZING**
**17**	**A: CYS96>ARG**	**1.16**	**DESTABILIZING**
**18**	**A: CYS96>LYS**	**1.52**	**DESTABILIZING**
**19**	**A: CYS96>GLY**	**1.7**	**DESTABILIZING**
**20**	**A: CYS96>PRO**	**5.02**	**DESTABILIZING**

### Determination of the crystal structure of FGF2 mutants

**Fig 3:**
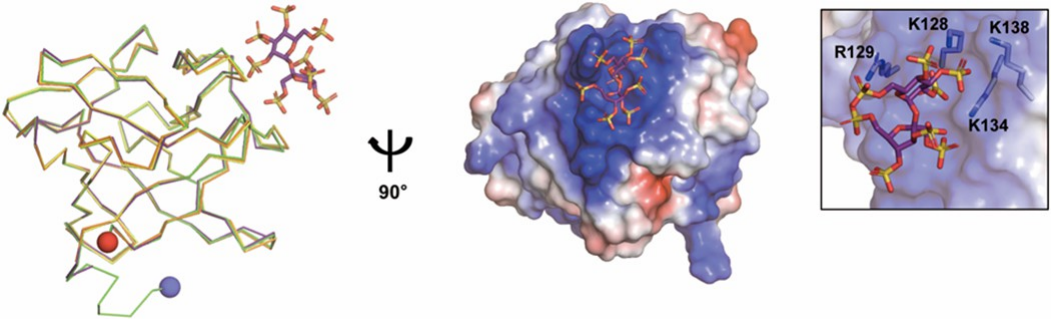
Structural comparison of FGF2-M1 and the FGF2-M2/SOS complex. *Left*, The superimposed Cα tracing of the FGF2 wild type (yellow; PDB code: 4FGF), the structure of the FGF2 mutant harboring C78S & C96S (orange; PDB code: 1BFG), FGF2-M1 (green), and the FGF2-M2/SOS complex (purple). The N- and C-terminus of FGF2-M1 are shown as blue and red spheres, respectively. *Right*, Surface representation with electrostatic potentials of FGF2-M2 with SOS (purple stick). The positively and negatively charged regions on the surface are colored in blue and red, respectively. In the box, the SOS-interacting residues of FGF2-M2 are shown as white sticks with labels. Nitrogen, oxygen, and sulfur atoms in the sticks are colored in blue, red, and yellow, respectively.

**Fig 4:**
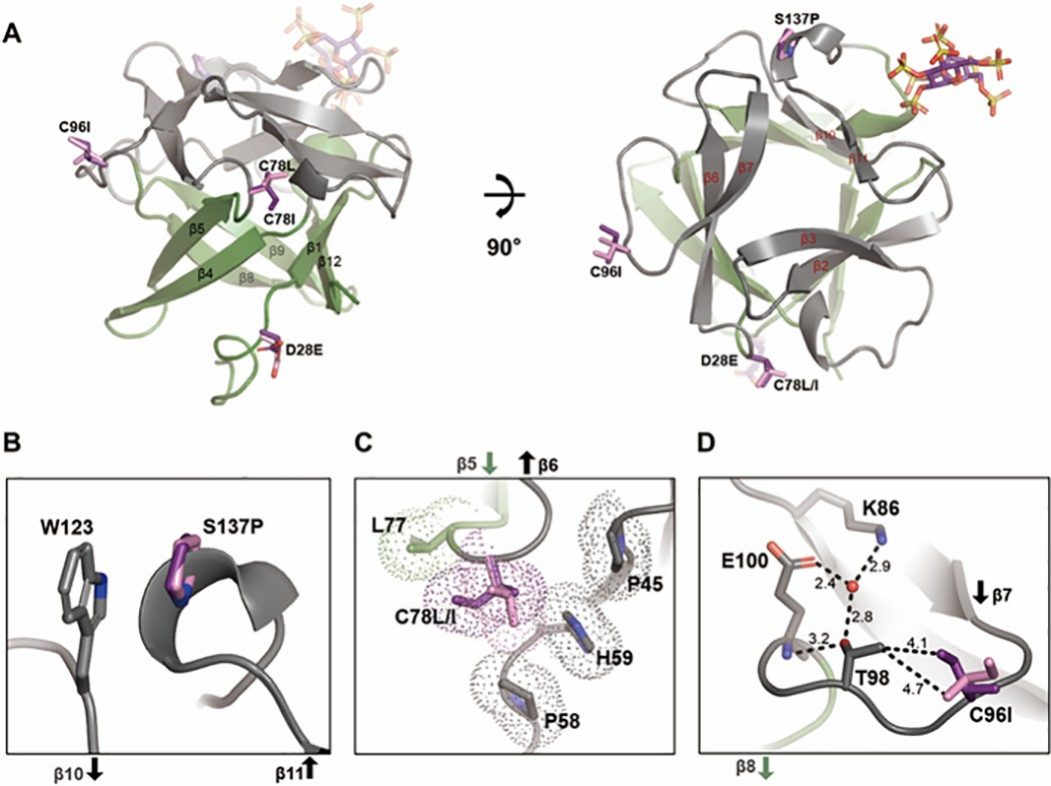
The mutation sites in the crystal structures of FGF2-M1 and FGF2-M2. (A) A side view & a top view of cartoon diagrams of FGF2 mutants labeled with each β strand in a β-barrel domain (green) and a triangular cap domain (gray). For brevity, only a cartoon diagram of the FGF2-M2/SOS complex is presented while the mutations in both structures of FGF2-M1 (pink sticks) and FGF2-M2 (purple sticks) are represented. (B-D) Close-up view of the mutation sites. The interacting residues with the mutation sites are shown as labeled sticks. The arrows indicate the β strands. Nitrogen, oxygen, and sulfur atoms in the sticks are colored blue, red, and yellow, respectively. (C) The surfaces of C78L, C78I, and interacting residues (P45 & P58 & H59 & L77) are represented as dots. (D) Interactions between C96I, T98, adjacent residues (K86 & E100), and a water molecule (a red sphere) are depicted by dashed lines with labeled distances (Å).

We successfully solved the high-resolution (1.40 Å and 1.48 Å, respectively) crystal structures of FGF2-M1 and the FGF2-M2/SOS complex (Fig 3 and S2 Table). The crystallization of FGF2-M1 was successful in the absence of SOS. On the other hand, SOS was used as an additive reagent to promote the crystallization of FGF2-M2 because it binds to the heparin-binding site of FGF2 [55]. The structures of FGF2-M1 and FGF2-M2 were superimposed to that of the wild type (PDB code: 4FGF) with a root-mean-square deviation (RMSD) of 0.336 Å and 0.328 Å for all Cα atoms, respectively. When superimposed onto the structure of the FGF2 mutant harboring two-point mutations (C78S & C96S; PDB code: 1BFG) [56], they exhibited the RMSD value of 0.256 Å and 0.275 Å for all Cα atoms, respectively (Fig 3). The RMSD value between the structures of the FGF2 mutants was 0.144 Å for all Cα atoms, indicating that the introduction of mutations did not affect the overall structure of FGF2. Both of them adopted the canonical β-trefoil fold, in which two sets of three β-hairpins are arranged with a pseudo-three-fold symmetry [57]. The β1–β12, β4–β5, and β8–β9 hairpins formed a β-barrel domain, which was covered with a triangular cap domain constructed by three other β-hairpins (β2–β3, β6–β7, and β10–β11) (Figs 3 and 4A). In the crystal structure of the FGF2-M2/SOS complex, negatively charged SOS was bound to the positively charged HSPG-binding site harboring K128, R129, K134, and K138 through extensive electrostatic interactions (Fig 3).

### Structural investigation into the mutation sites in FGF2 mutants

The S137P mutation was designed based on sequence comparison between human and whale FGF2s (Fig 1A). As shown in Fig 4B, P137 is located at the end of the β11 strand and contacts with the side-chain indole ring of W123, positioned at the beginning of the β10 strand. It has been established that the electron-rich π face of the indole ring forms a CH-π interaction with the partially positively charged hydrogen atoms adjacent to the carbonyl and amide nitrogen of P137 [58]. Therefore, S137P seems to improve the thermo-stability of FGF2 by anchoring the beginning and end of the β10–β11 hairpin such as a knot. Since the β10–β11 hairpin contains a heparin-binding site that is essential for the activity of FGF2, stabilization of the region is noteworthy (Fig 4B) [6, 7, 16, 59].

The D28E mutation is situated at the end of the N-terminal loop (residues 20–28 in the structure of FGF2-M1) independent from the β-trefoil fold of FGF2 (Fig 4A and S3A Fig). In crystal structure, the *B*-factor reflects how scattered the electron density is and how the atoms are arranged. To calculate the flexibility of the N-terminal loop of FGF2 mutants, the *B*-factor values for Cα atoms in the loop were normalized (*B*´ value) to the overall *B*-factor for all Cα atoms in the structures of the FGF2 mutants (S3A Fig). The *B*´ value of the N-terminal loop was 2.82 Å^2^, suggesting that the region could be structurally disordered and easily exposed to protease [50, 60]. This observation is also consistent with the fact that the N-terminal loop (residues 1–28) in the NMR structure of FGF2 (PDB code: 1BLA) is disordered and exhibits high mobility (S3A Fig) [61]. The N-terminal loop of FGF2 serves as the primary site for direct interaction with FGFR, initiating the signaling pathway [2, 8, 10]. In the crystal structure of the FGF2 mutant (C78S & C96S) and FGFR1c complex (PDB code: 1CVS), it was observed that the side chains of F17, K21, and Y24 in the N-terminal loop of FGF2 were found to interact with FGFR1c [8]. Accordingly, we constructed *in silico* model structure of the FGF2-M1/FGFR1c complex on the basis of the complex structure (PDB code: 1CVS). Structural analysis of the model structure showed that the side chain of D28E was at least 7.5 Å away from the surface of FGFR1c (S3B Fig), indicating that the mutation at position 28 only confers resistance to fragmentation of FGF2 without affecting its interaction with FGFR1c (Fig 2 and S3B Fig). Taken together, we could know that it was reasonable to introduce the D28E and S137P mutations in this region for structural stability and function.

The surface-exposed cysteines (C78 and C96 positioned within the β5–β6 loop and β7–β8 loop, respectively; Fig 4A) have been known to lead the oligomerization of FGF2 through intermolecular disulfide bond [37]. The structures of FGF2-M1 and FGF2-M2 showed that leucine or isoleucine at position 78 were sandwiched by hydrophobic interactions consisting of P45, P58, H59, and L77 (Fig 4C). In fact, free cysteine is recognized for its hydrophobic properties with a hydropathy scale of 2.5 and a respective volume of 108.5 Å^3^ [62, 63]. Thus, C78L and C78I seem to allow for better interactions with neighboring residues, which prefer a bit more hydrophobic and a bit larger amino acid; the hydropathy scales of leucine and isoleucine are 3.8 and 4.5, respectively, with their volumes of 166.7 Å^3^ [63]. As shown in Fig 4D, the C96I mutation formed a van der Waals interaction with the methyl group of T98 at a distance ranging from 4.1 Å to 4.7 Å. The hydroxyl group of T98 formed hydrogen bonds with the main chain amino (-NH) group of E100 and a water molecule at the distance of 3.2 Å and 2.8 Å, respectively. This water molecule further established two additional hydrogen bonds with the side chains of K86 and E100 (Fig 4D). The introduction of isoleucine at position 96 in the FGF2 mutants likely dictates the orientation of the T98 side-chain. Consequently, the hydrophobic replacements of surface-exposed cysteines enhanced the stability of FGF2, as this fostered extensively hydrophobic interactions with adjacent residues.

### Assessment of stability of FGF2 mutants

To investigate how susceptible FGF2 mutants are to thermal denaturation, the FGF2-containing solutions were incubated at 45 °C for 7 days. When proteins are denatured, they form aggregates. The heat-treated FGF2 solutions were centrifuged to remove protein aggregates, and then supernatants were analyzed using SDS-PAGE (S4 Fig). The thermal stability was assessed by the portion of soluble FGF2. As shown in S4 Fig, the soluble wild type disappeared after one day, while the D28E/S137P-harboring FGF2 mutants maintained soluble in a considerable amount for 7 days. Furthermore, FGF2-M1 and FGF2-M2 exhibited higher thermal stability than FGF2 D28E/S137P.

**Fig 5:**
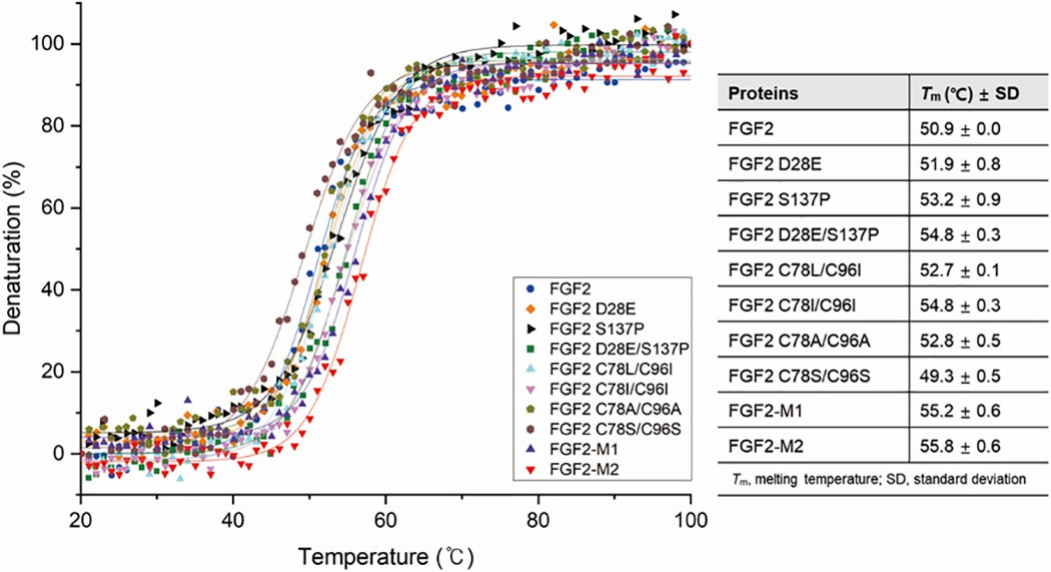
Thermal denaturation curve measured by CD experiments. Each graph displays the temperature-dependent spectral change at 228 nm. FGF2 and the mutants are represented by different colored lines and symbols, as shown in the right box of the figure.

For *T*_m_ value comparison among the FGF2 mutants, we monitored their thermal denaturation curves within the temperature range of 20–100 °C [57]. As depicted in Fig 5, the *T*_m_ values of FGF2 S137P (53.2 ± 0.9 °C) and FGF2 D28E/S137P (54.8 ± 0.3 °C) were higher than that of the wild type (50.9 ± 0.0 °C). Meanwhile, FGF2 D28E exhibited a similar *T*_m_ value of 51.9 ± 0.8 °C to the wild type. Regarding mutations involving surface-exposed cysteines (C78 and C96), the replacements with alanine or serine did not yield a significant enhancement in the thermal stability of FGF2. The *T*_m_ values of FGF2 mutants with C78A/C96A (52.8 ± 0.5 °C) and C78S/C96S (49.3 ± 0.5 °C) were slightly higher and lower, respectively, compared to that of the wild type. On the other hand, FGF2 mutants harboring C78L/C96I and C78I/C96I displayed *T*_m_ values of 52.7 ± 0.1 °C and 54.8 ± 0.3 °C, respectively. Consequently, the combination of four-point mutations in FGF2-M1 (55.2 ± 0.6 °C) and FGF2-M2 (55.8 ± 0.6 °C) yielded *T*_m_ values around 5 °C higher than that of the wild type (Fig 5). This suggests that the combined impact of these mutations synergistically contributes to enhancing the thermo-stability of FGF2 against heat-induced stress.

**Fig 6:**
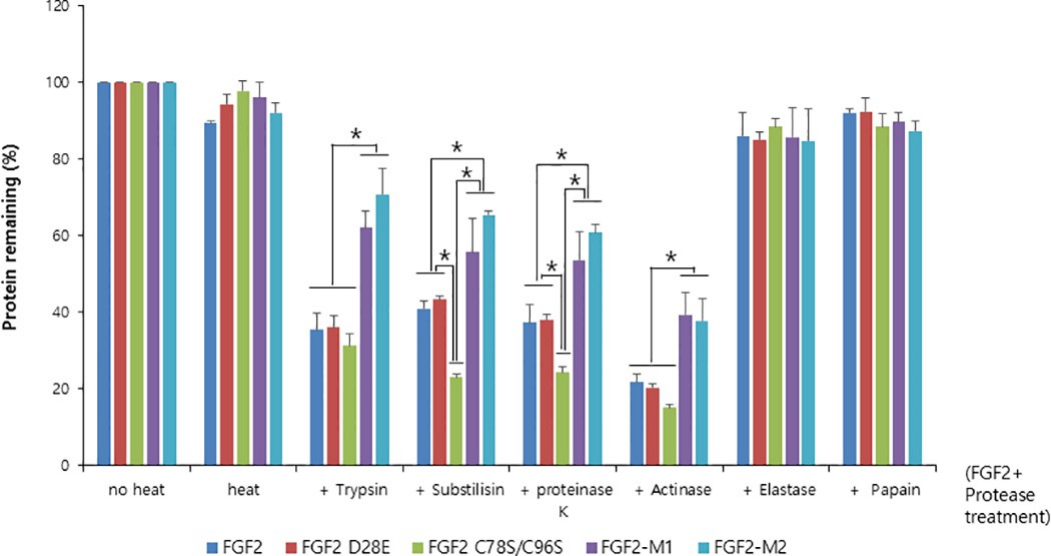
Comparison of protease digestion profiles of FGFs. Six different proteases were mixed with FGF2 variants (FGF2, FGF2 D28E, FGF2 C78S/C96S, FGF2-M1, and FGF2-M2). The protease reaction was conducted at 37 °C for 3 hours. The remaining proteins identified in the SDS-PAGE gel were quantified using the ImageJ program. The quantified values were presented as a bar graph. Cleaved proteins were excluded from quantification. The horizontal axis of the graph represents the following order: unincubated FGF2, incubated FGF2, trypsin, subtilisin, protease K, actinase, elastase, and papain. The vertical axis of the graph represents the percentage of protease digestion (N = 3, average ± SD). The statistical differences at each protease were performed by one-way ANOVA with Tukey’s post hoc test. Asterisk (*) indicates *P* < 0.05.

To evaluate the protease resistance of FGF2 mutants, the proteins were incubated for 3 hours at 37 °C using 12 proteases. The protease-treated FGF2s were centrifuged, and aggregates and cleaved peptides were excluded from the analysis. The FGF2 mutants showed differences in protease resistance when tested with trypsin (cleavage site: lysine or arginine, except when either is followed by proline), subtilisin (cleavage site: uncharged residue), proteinase K (cleavage site: aliphatic and aromatic amino acids), and actinase (cleavage site: glutamic or aspartic acid) out of the 12 proteases tested (S5 Fig). These four proteases, along with two other proteases—elastase (cleavage site: glycine, alanine, and valine) and papain (cleavage site: leucine or glycine)—which showed minor differences in digestion, were selected. SDS-PAGE analysis after treatment with these proteases was performed three times (Fig 6 and S4 Table). In protease resistance experiments, undigested FGF2 was visualized using an SDS-PAGE gel and presented as a bar graph. Also, in cases where the results of protease-treated FGF2 were statistically significant (*p* < 0.05), they were marked with an asterisk (*) in Fig 6. In the experiment, unincubated FGF2 and incubated FGF2 without protease were used as controls. FGF2 treated with elastase and papain did not show a significant statistical difference. However, results from trypsin and actinase treatments indicated that FGF2-M1 and FGF2-M2 exhibited higher protease resistance compared to FGF2, FGF2 D28E, and FGF2 C78S/C96S. Subtilisin and proteinase K treatment results revealed that FGF2 C78S/C96S displayed lower protease resistance than FGF2 and FGF2 D28E, while FGF2-M1 and FGF2-M2 showed enhanced protease resistance compared to FGF2 and FGF2 D28E. These results suggest that FGF2-M1 and FGF2-M2, which contain hydrophobic replacements for surface-exposed cysteines, exhibit improved protease resistance compared to the FGF2 wild type and a serine-substituted mutant.

**Fig 7:**
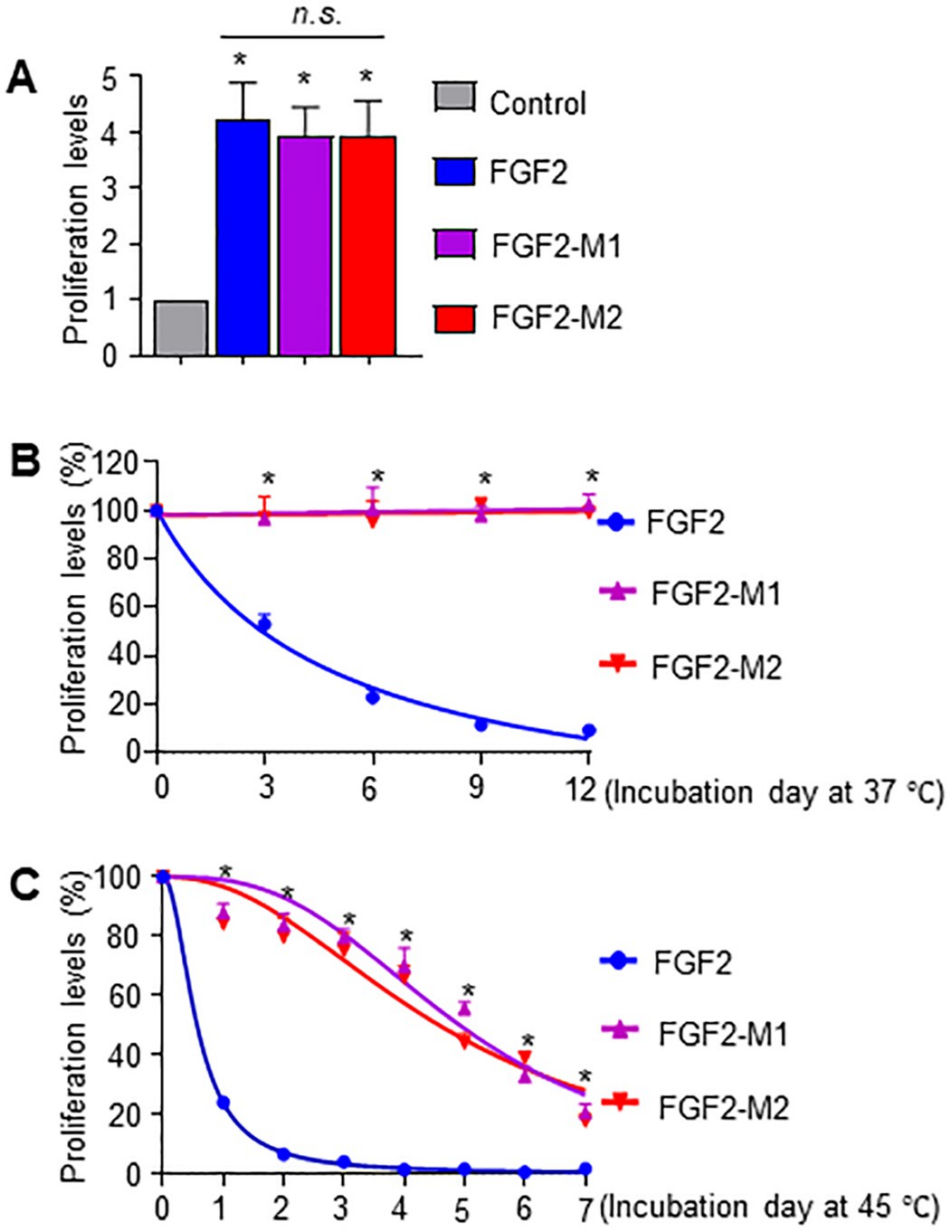
Cell proliferation activity of FGF2 mutants. (A) BALB3T3 cells were treated with 0.3 ng/mL FGF2s for 40 h, and then the proliferation assay was performed using cell counting kit-8. The proliferation levels of non-treated cells (Control) were set to 1, and that of the treated cells was calculated relative to this value (n = 4, average ± SD). The statistical differences were analyzed using the one-way ANOVA, followed by Tukey’s post hoc test. *P < 0.05 vs. Control. n.s., not significant. (B, C) After incubation of FGF2s at 37 °C (B) or 45 °C (C) for the indicated duration, their residual activity was determined using the cell proliferation assay. BALB3T3 cells were treated with 0.3 ng/mL FGF2s for 40 h, followed by the cell proliferation assay. The proliferation levels of non-incubated FGF2 at 37 °C (B) or 45 °C (C) were set to 100%, and that of the treated cells was calculated relative to this value (n = 3, average ± SD). Non-linear regression was performed using GraphPad Prism 9.4, and the best-fit non-linear regression curve is depicted. The statistical differences at each time point were calculated using one-way ANOVA followed by Tukey’s post hoc test. *P < 0.05. The dotted lines indicate half-levels of proliferation and their incubation day.

To confirm the thermal stability of the FGF2 mutants by biochemical methods, we used a cell proliferation assay in BALB3T3 cells to monitor residual biological activity after thermal incubation. The activities of FGF2-M1 and FGF2-M2 were similar to that of the wild type (Fig 7A and S5 Table), indicating that the mutation did not change the biological activity of FGF2. At 37 °C, the cell proliferation activity of the FGF2 wild type decreased with incubating duration, wherein the activities of FGF2-M1 and FGF2-M2 were maintained (Fig 7B and S5 Table). At 45 °C, the activity of the wild type almost disappeared after two days, but the activities of FGF2-M1 and FGF2-M2 were maintained at more than 80% (Fig 7C and S5 Table). The durations for reaching 50% residual activity of FGF2-M1 (4.92 day) and FGF2-M2 (4.58 day) were increased to 8.8-fold and 8.2-fold, respectively, compared to the wild type (0.56 day). These results suggest that the FGF2 mutants exhibit improved thermal stability while retaining their biological activities.

## Conclusion

Protein stability is a critical factor in pharmaceutical and industrial fields. Factors that affect protein stability include temperature, proteolytic cleavage, ionic strength, etc. Due to these factors and the distinct characteristics of each protein, it is difficult to increase stability through a common engineering strategy. Presenting various approaches to improve the stability of FGFs could have a positive impact on pharmaceutical and industrial fields. In particular, FGF2 plays diverse biological roles and has emerged as a potential therapeutic target for several diseases. However, its practical application has been hindered by its short half-life. Previous research on FGF2 predominantly focused on enhancing its thermo-stability for applications in cell cultivation media such as FGF2-G3 including nine-point mutations [12, 13, 16, 17]. However, we paid attention to showing the different strategies. In our approach, novel mutation sites (S137P and D28E) were introduced into FGF2 by utilizing genomic data from marine organisms and demonstrating enhanced resistance to glutathione. Additionally, the surface-exposed cysteines of FGF2 were replaced with energetically optimized hydrophobic residues (C78L, C78I, and C96I) through *in silico* calculations. We could figure out the structural effects of each mutation on improving the stability of the FGF2 mutants (Fig 4). Although 22 structures of FGF2 have been deposited in the Protein Data Bank, there was no report describing how mutations contributed to the stability of FGF2 at the atomic level. Thus, this study could provide structural insights into engineering strategies to improve the thermal stability of FGF2.

To enhance the therapeutic potentials of protein, genomic data from organisms residing in diverse habitats, like marine environments, have been utilized [25]. Therefore, we investigated whether the thermal stability of FGF2 can be improved by adopting marine genome information. Initial findings revealed that human and whale FGF2s had highly similar sequence identities, except for only two residues at positions 121 and 137 among 155 residues. Subsequently, the S137P mutation demonstrated an improvement in FGF2’s thermo-stability, as determined by SDS-PAGE and CD experiments (Figs 1B, 1C and 5). It was also identified through the crystal structures of FGF2-M1 and FGF2-M2 (Fig 4); the substituted proline residue at position 137 forms CH-π interactions with W123 to stabilize the β10–β11 hairpin, a portion of the heparin-binding site of FGF2. Since HSPG is essential for the activity of FGF2, it is important to stabilize the heparin binding site. For this purpose, strategies have been developed in clinical applications to maintain the activity of FGF2 by adding heparin or conjugating it with synthetic glycosaminoglycans (GAGs) [14]. It has also been reported that GAGs, including heparin, contribute to the stabilization of FGF [64, 65]. Notably, heparin binding to FGF2 increased *T*_m_ by 22.2 °C [66]. As shown in the cell proliferation assay (Fig 7), the activities of FGF2-M1 and FGF2-M2 were maintained 8.2~8.8-fold longer than that of the wild type in the presence of heparin. Taken together, these results suggest the potential of the FGF2 variants for further optimization and exploration of synergistic effects with other stabilizing agents or modifications.

Disulfide bonds play an important role in protein folding and stability by pairing cysteines [67]; however, mismatching cysteines can lead to misfolding and aggregation [68]. Since two cysteines on the FGF2 surface caused protein oligomerization [37], we treated FGF2 with glutathione to reduce the oligomerization. However, the addition of GSH or GSSG fragmented FGF2 into a fixed pattern, which was cleaved after the aspartic acid residues. The introduction of D28E made FGF2 resistant to cleavage and affected its thermo-stability in CD results as well (Fig 5). In addition, the double mutation of D28E/S137P yielded a more effective enhancement in the thermo-stability compared to the wild type or the introductions of each single mutation, indicating a synergistic effect of the point mutations. Substitution of cysteine with serine or alanine, which has structural similarity without the chemical properties of cysteine, has generally been employed for structural or functional studies of proteins. It has been reported that the mutation of Cys → Ser also stabilized FGF2 by preventing its aggregation [38]. However, our strategy was not only to prevent aggregation by surface-exposed cysteines but also to energetically optimize adjacent residues. The residues with the best results were selected by using SDM and Discovery Studio 2019. It suggested that leucine or isoleucine at positions 78 and 96 were more energetically stabilized than the introduction of alanine or serine (Tables 2 and 3). The modification of C78 and C96 with leucine or isoleucine did not form oligomers and exhibited higher *T*_m_ values than other FGF2 mutants, supported by SDS-PAGE and the CD analysis (Fig 5 and S4 Fig). From a structural perspective, C78L, C78I, and C96I of the FGF2 mutants extensively form hydrophobic interactions with adjacent residues (Fig 4C and 4D). Therefore, the Cys → Ala or Ser substitutions showed no better improvement in thermal stability of FGF2 than our hydrophobic replacements. Notably, our structural studies and CD measurements explained the reason why the hydrophobic replacements showed more effectiveness in enhancing the stability of FGF2 than other mutations (Figs 4 and 5). These results suggest that the energy optimization moiety may vary depending on the *in silico* method and structural analysis should be performed together.

Consequently, FGF2-M1 and FGF2-M2 harboring the four-point mutations were obtained by combining bioinformatics, molecular thermodynamics, and molecular modeling. They exhibited higher thermo-stability than other FGF2s, which was further supported by the results of SDS-PAGE, ~5 °C higher *T*_m_ values, and 8.2 ~ 8.8-fold longer cell proliferation activity at 45 °C than the FGF2 wild type. Additionally, FGF2-M1 and FGF2-M2 with introduced hydrophobic residues showed improved resistance to the limited proteolysis. FGF2 mutants were reacted with 12 different proteases for 3 hours. FGF2-M1 and FGF2-M2 showed enhanced resistance to four proteases (trypsin, subtilisin, protease K, and actinase), while the wild type, FGF2 D28E, and FGF2 C78S/C96S showed lower resistance to these proteases (Fig 6). This means that FGF2-M1 and FGF2-M2, which contain novel mutation sites and hydrophobic replacements for cysteines, show higher resistance against protease. Combined with the structural descriptions, our strategies not only contributed to improved thermal stability but also exhibited more enhanced resistance to protease compared to the wild type. Additionally, FGF2-M1 and FGF2-M2 also affected protein stability and increased protein yield (S6 Fig). The yields of FGF2, FGF2-M1, and FGF2-M2 were 27.48%, 46%, and 36.13% respectively. Thus, these findings may help to further improve our understanding of developing FGF2 in pharmaceutical and industrial applications.

## Supporting information

S1 Table Value of reverse-phase HPLC in Fig 1. (DOCX)

S2 Table Data collection and refinement of FGF2 crystals. (DOCX)

S3 Table The values of *T*_m_ for the FGF2 wild type and mutants showed in Fig 5. (DOCX)

S4 Table The values of protease resistance for the FGF2 wild type and mutants showed in Fig 6. (DOCX)

S5 Table The values of protease resistance for the FGF2s showed in Fig 7. (DOCX)

S1 Fig N-terminal sequences of pFGF2 and FGF2. pQE80 and pET17b for expressing pFGF2 (residue 1–155) and FGF2 (residue 10–155), respectively, are indicated to the left of the corresponding sequence. The residue number is shown above the sequence.
(TIF)

S2 Fig Analysis of N-terminus of FGF2 fragments. N-terminal sequences of FGF2 protein fragments after the addition of glutathione were determined by GelNrich-coupled mass spectrometry. The sequence in the top black box represents the complete sequence of pFGF2. FGF2 cloned into the pQE80 vector has 25 additional sequences, including a His tag at the N-terminus. The graph on the right contains sequence information confirming the N-terminal sequence using the GenNrich method. The GenNrich method enriches N-terminal peptides following trypsin digestion of a protein or a fragment (red bar). The table indicates the semi-tryptic peptides identified. In particular, the PSM values of the peptide starting at amino acid 15 (band 2) (GGSGAFPPGHFKDPKR) and the peptide starting at amino acid 57 (PHIKLQLQAEER) were the most abundant. The starting sequences of bands 2 and 3 identified in the SDS-PAGE gel are indicated by red arrows in the sequence above the graph on the right.
(TIF)

S3 Fig Structural analysis of the D28E mutation in the N-terminal loop. (A) Superimposition between the FGF2-M1 structure and the NMR structure (PDB code: 1BLA). Their N-terminus are shown as spheres. For clarity, only the N-terminal loop (residues 1–29) of the NMR structure is shown as black, while the full structure of FGF2-M1 is shown with *B*-factor representation. The color spectrum (red to blue) represents a range of *B*-factor values (43.63 to 9.02), which corresponds to a range of *B’*-factor values (3.38 to -1.28). The D28E mutation in FGF2-M1 is shown as a pink stick with a label. (B) Superposed model structure of the FGF2-M1/FGFR1c complex. From the template (PDB code: 1CVS), D28 is shown as a white stick with a label and FGFR1c is also depicted in a grey surface representation. The distance between the E28 side-chain and the surface of FGFR1c is indicated by a black dashed line.
(TIF)

S4 Fig Thermal stability of FGF2 mutants. The FGF2 mutants were incubated at 45 °C for 7 days and checked for thermal stability using 15% SDS-PAGE.
(TIF)

S5 Fig Comparison of protease resistance of FGF2 mutants. FGF2 variants (FGF2, FGF2 D28E, FGF2 C78S/C96S, FGF2-M1, FGF2-M2) were mixed with 12 different proteases and quantified using SDS-PAGE gel. Protease reactions were performed at 37 °C for 3 h. M: protein size marker, Lanes 1 and 2 represent unincubated and incubated protein samples, respectively. Lines 3: trypsin, 4: elastase, 5: chymotrypsin, 6: papain, 7: subtilisin, 8: endoproteinase Glu-C, 9: protease K, 10: clostripain (endoproteinase-Arg-C), 11: pepsin, 12: thermolysin, 13: bromelain, 14: actinase.
(TIF)

S6 Fig Comparison of FGF2 yield. Calculation of (A) FGF2, (B) FGF2-M1, and (C) FGF2-M2 concentrations in supernatants of crude extracts by western blot. (D) Using the Western blot method, I calculated the standard curves for S6A-S6C Fig. To determine the peak levels of FGF2, I used the supernatant diluted 1/24 from the lysed cells. (E) production comparison table for each stage of FGF2s purified in two stages.
(TIF)
