# Supplementary material for: Structural and biochemical investigation into stable FGF2 mutants with novel mutation sites and hydrophobic replacements for surface-exposed cysteines
Source: PLoS One. 2024 Sep 5;19(9):e0307499. doi: 10.1371/journal.pone.0307499 (PMC11376533; doi:10.1371/journal.pone.0307499)
Supplement: S1 Table — (DOCX) [file pone.0307499.s001.docx]

**S1 Table. Data collection and refinement of FGF2 crystals**

|  | **FGF2-M1** | **FGF2-M2/SOS** |
| --- | --- | --- |
| **PDB code** | 8HU7 | 8HUE |
| **Data collection** |  |  |
| **Diffraction source** | PAL/PLS beamline 11C | |
| **Wavelength (Å)** | 0.97941 | |
| **Rotation range per image (°)** | 0.5 | 1 |
| **Total rotation range (°)** | 360 | 360 |
| **Exposure time per image (s)** | 0.5 | 0.5 |
| **Space group** | P3_1_21 | P2_1_ |
| **a, b, c (Å)/α, β, γ (°)** | a = b = 54.375, c = 97.012/  γ = 120 | 51.38, 54.45, 71.97/  β = 101.459 |
| **Resolution range (Å) ^a^** | 50-1.40 (1.42-1.40) | 50-1.48 (1.51-1.48) |
| **Total No. of reflections** | 484810 | 365816 |
| **No. of unique reflections** | 33205 (1638) | 64285 (3196) |
| **Completeness (%) ^a^** | 99.8 (99.3) | 98.3 (97.4) |
| **CC (1/2) (%) ^a^** | 100.5 (83.7) | 98.6 (82.5) |
| **Redundancy ^a^** | 14.6 (9.4) | 5.7 (4.7) |
| **Mean I/σ (I) ^a^** | 64.3 (5.2) | 26.1 (3.9) |
| **R_merge_ (%) ^a, b^** | 11.3 (45.8) | 10.5 (38.3) |
| **Refinement statistics** |  |  |
| **Resolution range (Å) ^a^** | 47.09-1.40 (1.45-1.40) | 45.48-1.48 (1.53-1.48) |
| **No. of reflections of working set ^a^** | 31181 (3023) | 62269 (6016) |
| **No. of reflections of test set ^a^** | 2003 (198) | 1999 (193) |
| **No. of atoms** | 1223 | 3618 |
| **Protein** | 1085 | 3148 |
| **Water** | 138 | 360 |
| **Ligand** | - | 110 |
| **Average B value (**$\text{Å}^{\mathbf{2}}$**)** |  |  |
| **Protein** | 18.5 | 15.9 |
| **Water** | 28.7 | 24.6 |
| **Ligand** | - | 34.9 |
| **R_work_/R_free_ (%) ^a, c^** | 18.51 (21.73)/21.27 (22.67) | 17.91 (21.77)/19.56 (27.72) |
| **R.m.s deviations ^d^** |  |  |
| **Bond length (Å)** | 0.015 | 0.014 |
| **Bond angles (°)** | 1.41 | 1.90 |

^a^ The number in parentheses is for the outer shell.

^b^ *R_merge_* =$\sum_{hkl} \sum_{i} \left| I_{i}\left( hkl \right)-\left\langle I(hkl) \right\rangle\right|/\sum_{hkl} \sum_{i} I_{i}(hkl)$, where $I_{i}(hkl)$ is the intensity of observed reﬂection$hkl$ and $\left\langle I(hkl) \right\rangle$ is the mean intensity of symmetry-equivalent reﬂections.

^c^ *R* = $\sum\left| F_{o}-F_{c} \right|/\sum F_{o}$, where $F_{o}=F_{p}$, and $F_{c}$ is the calculated protein structure factor from the atomic model. *R*_free_ was calculated with 10% of the reflections.

^d^ R.m.s. deviations in bond length and angles are the deviations from ideal values.
