# Supplementary material for: Structural and biochemical investigation into stable FGF2 mutants with novel mutation sites and hydrophobic replacements for surface-exposed cysteines
Source: PLoS One. 2024 Sep 5;19(9):e0307499. doi: 10.1371/journal.pone.0307499 (PMC11376533; doi:10.1371/journal.pone.0307499)
Supplement: S2 Table — (DOCX) [file pone.0307499.s002.docx]

**S2 Table. Value of reverse-phase HPLC in Fig 1.**

|  | **Peak area** | | | **Percentage (%)** | | |
| --- | --- | --- | --- | --- | --- | --- |
|  | **0 day** | **2 day** | **6 day** | **0 day** | **2 day** | **6 day** |
| **Wild** | 411.0 | 71.6 | 0.0 | 100.0 | 17.4 | 0.0 |
| **T121S** | 377.0 | 82.9 | 0.0 | 100.0 | 22.0 | 0.0 |
| **S137P** | 559.0 | 212.2 | 39.6 | 100.0 | 38.0 | 7.1 |
| **T121S/S137P** | 656.5 | 158.3 | 0.0 | 100.0 | 24.1 | 0.0 |
