# Supplementary material for: Structural and biochemical investigation into stable FGF2 mutants with novel mutation sites and hydrophobic replacements for surface-exposed cysteines
Source: PLoS One. 2024 Sep 5;19(9):e0307499. doi: 10.1371/journal.pone.0307499 (PMC11376533; doi:10.1371/journal.pone.0307499)
Supplement: S3 Table — (DOCX) [file pone.0307499.s003.docx]

**S3 Table. The values of *T*_m_ for the FGF2 wild type and mutants showed in Fig 5.**

| **Protein** | ***T*_m_ (℃)** | | | | **Standard deviation (SD; ℃)** |
| --- | --- | --- | --- | --- | --- |
|  | **1st** | **2nd** | **3rd** | **Mean** |  |
| **FGF2**  **(Wild type)** | 50.87 | 50.97 | 50.97 | 50.9 | 0.0 |
| **FGF2 D28E** | 52.35 | 52.51 | 50.74 | 51.9 | 0.8 |
| **FGF2 S137P** | 53.58 | 53.73 | 52.17 | 53.2 | 0.9 |
| **FGF2 D28E/S137P** | 55.13 | 54.76 | 54.44 | 54.8 | 0.3 |
| **FGF2 C78L/C96I** | 52.70 | 52.54 | 52.79 | 52.7 | 0.1 |
| **FGF2 C78I/C96I** | 54.76 | 54.59 | 55.18 | 54.8 | 0.3 |
| **FGF2 C78A/C96A** | 52.20 | 52.76 | 53.46 | 52.8 | 0.5 |
| **FGF2 C78S/C96S** | 48.71 | 49.27 | 50.04 | 49.3 | 0.5 |
| **FGF2-M1** | 54.71 | 54.70 | 56.07 | 55.2 | 0.6 |
| **FGF2-M2** | 55.31 | 55.34 | 56.70 | 55.8 | 0.6 |
