# Supplementary material for: Structural and biochemical investigation into stable FGF2 mutants with novel mutation sites and hydrophobic replacements for surface-exposed cysteines
Source: PLoS One. 2024 Sep 5;19(9):e0307499. doi: 10.1371/journal.pone.0307499 (PMC11376533; doi:10.1371/journal.pone.0307499)
Supplement: S4 Table — (DOCX) [file pone.0307499.s004.docx]

**S4 Table.** **The values of protease resistance for the FGF2 wild type and mutants showed in Fig 6.**

|  | **FGF2** | | | | |
| --- | --- | --- | --- | --- | --- |
|  | **1st** | **2nd** | **3rd** | **Average** | **Standard deviation** |
| **no heat** | 100 | 100 | 100 | 100.0 | 0.0 |
| **heat** | 89 | 89 | 90 | 89.3 | 0.6 |
| **+ Trypsin** | 31 | 40 | 35 | 35.3 | 4.5 |
| **+ Substilisin** | 41 | 43 | 39 | 41.0 | 2.0 |
| **+ proteinase K** | 32 | 39 | 41 | 37.3 | 4.7 |
| **+ Actinase E** | 21 | 24 | 20 | 21.7 | 2.1 |
| **+ Elastase** | 88 | 79 | 91 | 86.0 | 6.2 |
| **+ Papain** | 91 | 92 | 93 | 92.0 | 1.0 |
|  |  |  |  |  |  |
|  | **FGF2 D28E** | | | | |
|  | **1st** | **2nd** | **3rd** | **Average** | **Standard deviation** |
| **no heat** | 100 | 100 | 100 | 100.0 | 0.0 |
| **heat** | 97 | 95 | 91 | 94.3 | 3.1 |
| **+ Trypsin** | 39 | 38 | 31 | 36.0 | 4.4 |
| **+ Substilisin** | 45 | 42 | 43 | 43.3 | 1.5 |
| **+ proteinase K** | 38 | 37 | 39 | 38.0 | 1.0 |
| **+ Actinase E** | 20 | 22 | 19 | 20.3 | 1.5 |
| **+ Elastase** | 89 | 83 | 83 | 85.0 | 3.5 |
| **+ Papain** | 92 | 90 | 95 | 92.3 | 2.5 |
|  |  |  |  |  |  |
|  | **FGF2 C78S/C96S** | | | | |
|  | **1st** | **2nd** | **3rd** | **Average** | **Standard deviation** |
| **no heat** | 100 | 100 | 100 | 100.0 | 0.0 |
| **heat** | 95 | 98 | 100 | 97.7 | 2.5 |
| **+ Trypsin** | 32 | 34 | 28 | 31.3 | 3.1 |
| **+ Substilisin** | 24 | 23 | 22 | 23.0 | 1.0 |
| **+ proteinase K** | 23 | 26 | 24 | 24.3 | 1.5 |
| **+ Actinase E** | 14 | 15 | 16 | 15.0 | 1.0 |
| **+ Elastase** | 89 | 86 | 90 | 88.3 | 2.1 |
| **+ Papain** | 88 | 85 | 87 | 86.5 | 2.1 |
|  |  |  |  |  |  |
|  | **FGF2-M1** | | | | |
|  | **1st** | **2nd** | **3rd** | **Average** | **Standard deviation** |
| **no heat** | 100 | 100 | 100 | 100.0 | 0.0 |
| **heat** | 96 | 92 | 100 | 96.0 | 4.0 |
| **+ Trypsin** | 64 | 65 | 57 | 62.0 | 4.4 |
| **+ Substilisin** | 63 | 58 | 46 | 55.7 | 8.7 |
| **+ proteinase K** | 51 | 62 | 48 | 53.7 | 7.4 |
| **+ Actinase E** | 37 | 46 | 35 | 39.3 | 5.9 |
| **+ Elastase** | 88 | 92 | 77 | 85.7 | 7.8 |
| **+ Papain** | 87 | 91 | 91 | 89.7 | 2.3 |
|  |  |  |  |  |  |
|  | **FGF2-M2** | | | | |
|  | **1st** | **2nd** | **3rd** | **Average** | **Standard deviation** |
| **no heat** | 100 | 100 | 100 | 100.0 | 0.0 |
| **heat** | 93 | 94 | 89 | 92.0 | 2.6 |
| **+ Trypsin** | 63 | 76 | 73 | 70.7 | 6.8 |
| **+ Substilisin** | 64 | 66 | 66 | 65.3 | 1.2 |
| **+ proteinase K** | 59 | 60 | 63 | 60.7 | 2.1 |
| **+ Actinase E** | 31 | 42 | 40 | 37.7 | 5.9 |
| **+ Elastase** | 89 | 90 | 75 | 84.7 | 8.4 |
| **+ Papain** | 90 | 87 | 85 | 87.3 | 2.5 |
