# Supplementary material for: Structural and biochemical investigation into stable FGF2 mutants with novel mutation sites and hydrophobic replacements for surface-exposed cysteines
Source: PLoS One. 2024 Sep 5;19(9):e0307499. doi: 10.1371/journal.pone.0307499 (PMC11376533; doi:10.1371/journal.pone.0307499)
Supplement: S5 Table — (DOCX) [file pone.0307499.s005.docx]

**S5 Table. The values of protease resistance for the FGF2s showed in Fig 7.**

| **The values of Figure 7A.** | | | | | | |
| --- | --- | --- | --- | --- | --- | --- |
|  | **set1** | **set2** | **set3** | **set4** | **average** | **SD** |
| **Control** | 1.0 | 1.0 | 1.0 | 1.0 | **1.0** | **0.00** |
| **FGF2** | 4.7 | 4.9 | 3.7 | 3.6 | **4.2** | **0.66** |
| **FGF2-M1** | 4.5 | 4.2 | 3.3 | 3.6 | **3.9** | **0.53** |
| **FGF2-M2** | 4.3 | 4.6 | 3.3 | 3.4 | **3.9** | **0.65** |
| **The values of Figure 7B.** | | | | | | |
|  |  | **set1** | **set2** | **set3** | **average** | **SD** |
| **FGF2** | day0 | 100.0 | 100.0 | 100.0 | **100.0** | **0.0** |
|  | day3 | 57.5 | 52.5 | 49.3 | **53.1** | **4.1** |
|  | day6 | 21.6 | 26.4 | 20.3 | **22.8** | **3.2** |
|  | day9 | 13.5 | 11.4 | 9.7 | **11.5** | **1.9** |
|  | day12 | 11.2 | 7.5 | 9.3 | **9.3** | **1.9** |
| **FGF2-M1** | day0 | 100.0 | 100.0 | 100.0 | **100.0** | **0.0** |
|  | day3 | 97.7 | 97.5 | 95.0 | **96.8** | **1.5** |
|  | day6 | 91.6 | 100.0 | 109.8 | **100.5** | **9.1** |
|  | day9 | 93.4 | 100.5 | 100.4 | **98.1** | **4.1** |
|  | day12 | 100.0 | 107.4 | 100.0 | **102.5** | **4.3** |
| **FGF2-M2** | day0 | 100.0 | 100.0 | 100.0 | **100.0** | **0.0** |
|  | day3 | 92.9 | 91.2 | 107.2 | **97.1** | **8.8** |
|  | day6 | 91.3 | 89.6 | 105.2 | **95.4** | **8.6** |
|  | day9 | 102.7 | 101.0 | 105.5 | **103.1** | **2.3** |
|  | day12 | 99.9 | 99.1 | 98.6 | **99.2** | **0.6** |
| **The values of Figure 7C.** | | | | | | |
|  |  | **set1** | **set2** | **set3** | **average** | **SD** |
| **FGF2** | day0 | 100.0 | 100.0 | 100.0 | **100.0** | **0.00** |
|  | day1 | 27.5 | 22.7 | 21.9 | **24.0** | **3.02** |
|  | day2 | 5.9 | 7.5 | 6.1 | **6.5** | **0.87** |
|  | day3 | 4.5 | 4.3 | 3.2 | **4.0** | **0.69** |
|  | day4 | 0.0 | 3.6 | 0.6 | **1.4** | **1.95** |
|  | day5 | 2.4 | 2.4 | 0.0 | **1.6** | **1.39** |
|  | day6 | 0.0 | 1.5 | 0.0 | **0.5** | **0.85** |
|  | day7 | 0.0 | 4.9 | 0.2 | **1.7** | **2.77** |
| **FGF2-M1** | day0 | 100.0 | 100.0 | 100.0 | **100.0** | **0.00** |
|  | day1 | 79.2 | 89.4 | 86.3 | **85.0** | **5.26** |
|  | day2 | 75.9 | 85.9 | 88.9 | **83.6** | **6.82** |
|  | day3 | 74.9 | 79.1 | 84.7 | **79.6** | **4.89** |
|  | day4 | 58.8 | 71.0 | 79.8 | **69.9** | **10.56** |
|  | day5 | 51.8 | 59.5 | 55.2 | **55.5** | **3.86** |
|  | day6 | 28.0 | 38.7 | 31.7 | **32.8** | **5.48** |
|  | day7 | 15.4 | 25.7 | 20.0 | **20.4** | **5.16** |
| **FGF2-M2** | day0 | 100.0 | 100.0 | 100.0 | **100.0** | **0.00** |
|  | day1 | 79.1 | 82.5 | 86.0 | **82.5** | **3.49** |
|  | day2 | 73.4 | 73.5 | 92.6 | **79.8** | **11.03** |
|  | day3 | 66.4 | 72.4 | 86.5 | **75.1** | **10.29** |
|  | day4 | 58.1 | 71.0 | 69.0 | **66.0** | **6.95** |
|  | day5 | 37.5 | 47.3 | 47.2 | **44.0** | **5.61** |
|  | day6 | 37.8 | 39.8 | 39.3 | **39.0** | **1.05** |
|  | day7 | 14.5 | 21.7 | 17.2 | **17.8** | **3.59** |
