# Supplementary figures and images for: Structural and biochemical investigation into stable FGF2 mutants with novel mutation sites and hydrophobic replacements for surface-exposed cysteines
Source: PLoS One. 2024 Sep 5;19(9):e0307499. doi: 10.1371/journal.pone.0307499 (PMC11376533; doi:10.1371/journal.pone.0307499)

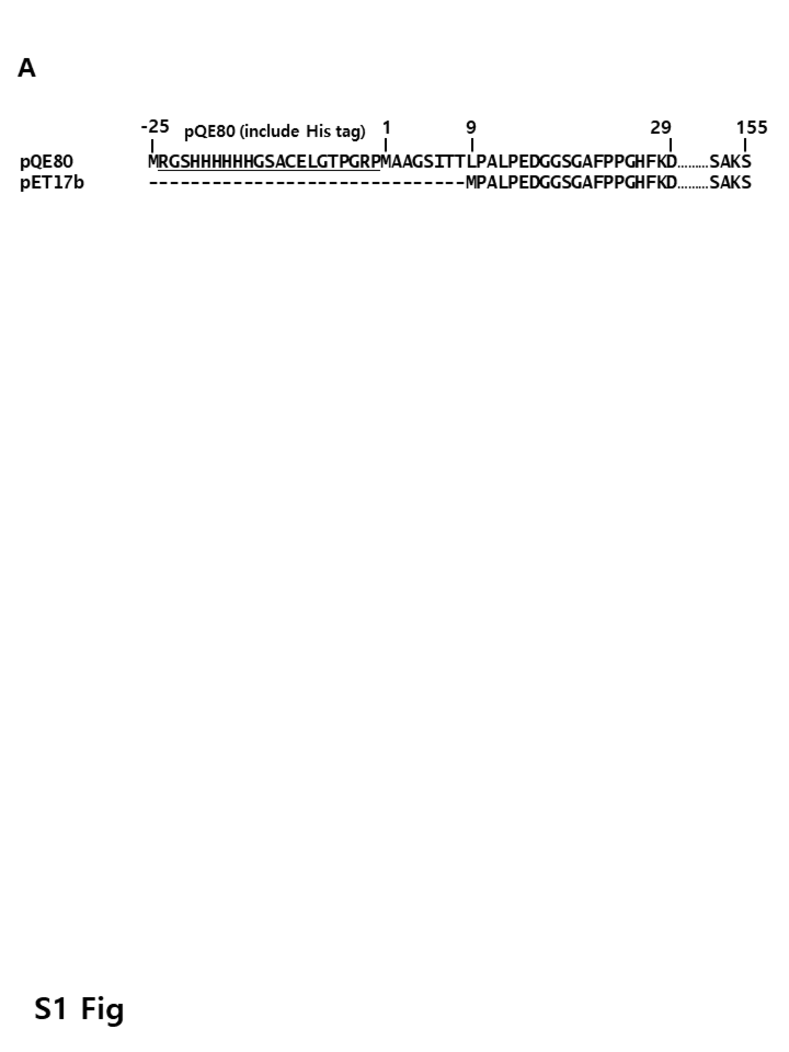

Supplement: S1 Fig — pQE80 and pET17b for expressing pFGF2 (residue 1–155) and FGF2 (residue 10–155), respectively, are indicated to the left of the corresponding sequence. The residue number is shown above the sequence. (TIF) [file pone.0307499.s006.tif]

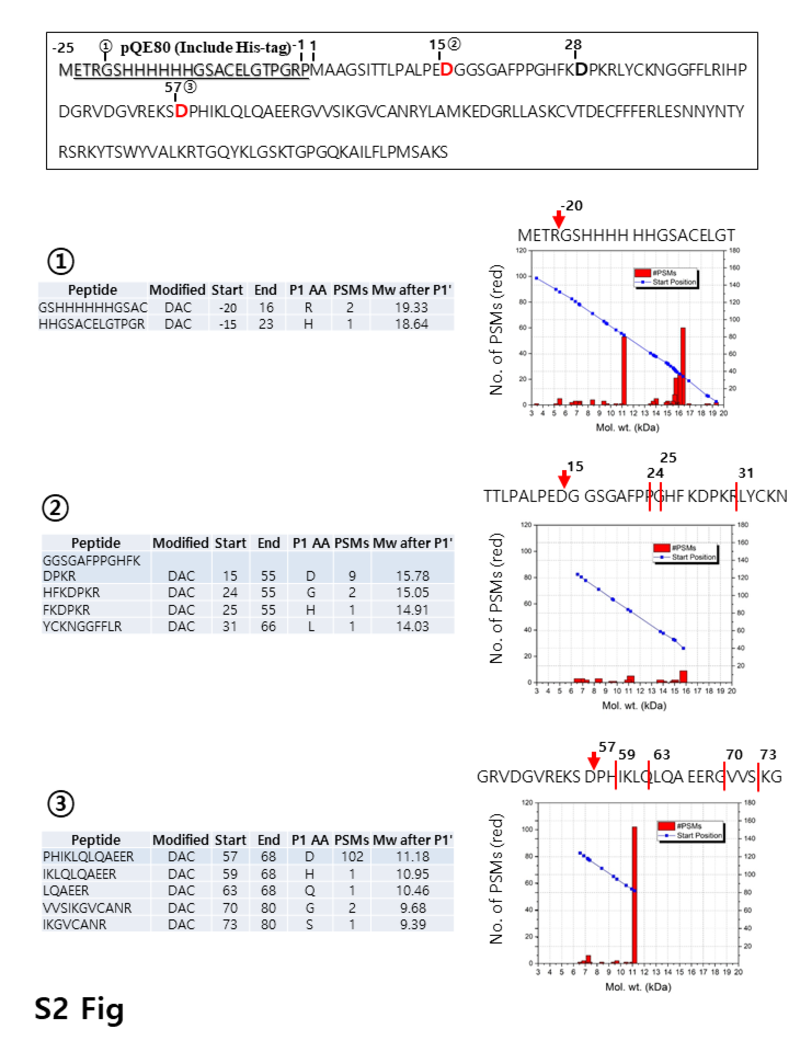

Supplement: S2 Fig — N-terminal sequences of FGF2 protein fragments after the addition of glutathione were determined by GelNrich-coupled mass spectrometry. The sequence in the top black box represents the complete sequence of pFGF2. FGF2 cloned into the pQE80 vector has 25 additional sequences, including a His tag at the N-terminus. The graph on the right contains sequence information confirming the N-terminal sequence using the GenNrich method. The GenNrich method enriches N-terminal peptides following trypsin digestion of a protein or a fragment (red bar). The table indicates the semi-tryptic peptides identified. In particular, the PSM values of the peptide starting at amino acid 15 (band 2) (GGSGAFPPGHFKDPKR) and the peptide starting at amino acid 57 (PHIKLQLQAEER) were the most abundant. The starting sequences of bands 2 and 3 identified in the SDS-PAGE gel are indicated by red arrows in the sequence above the graph on the right. (TIF) [file pone.0307499.s007.tif]

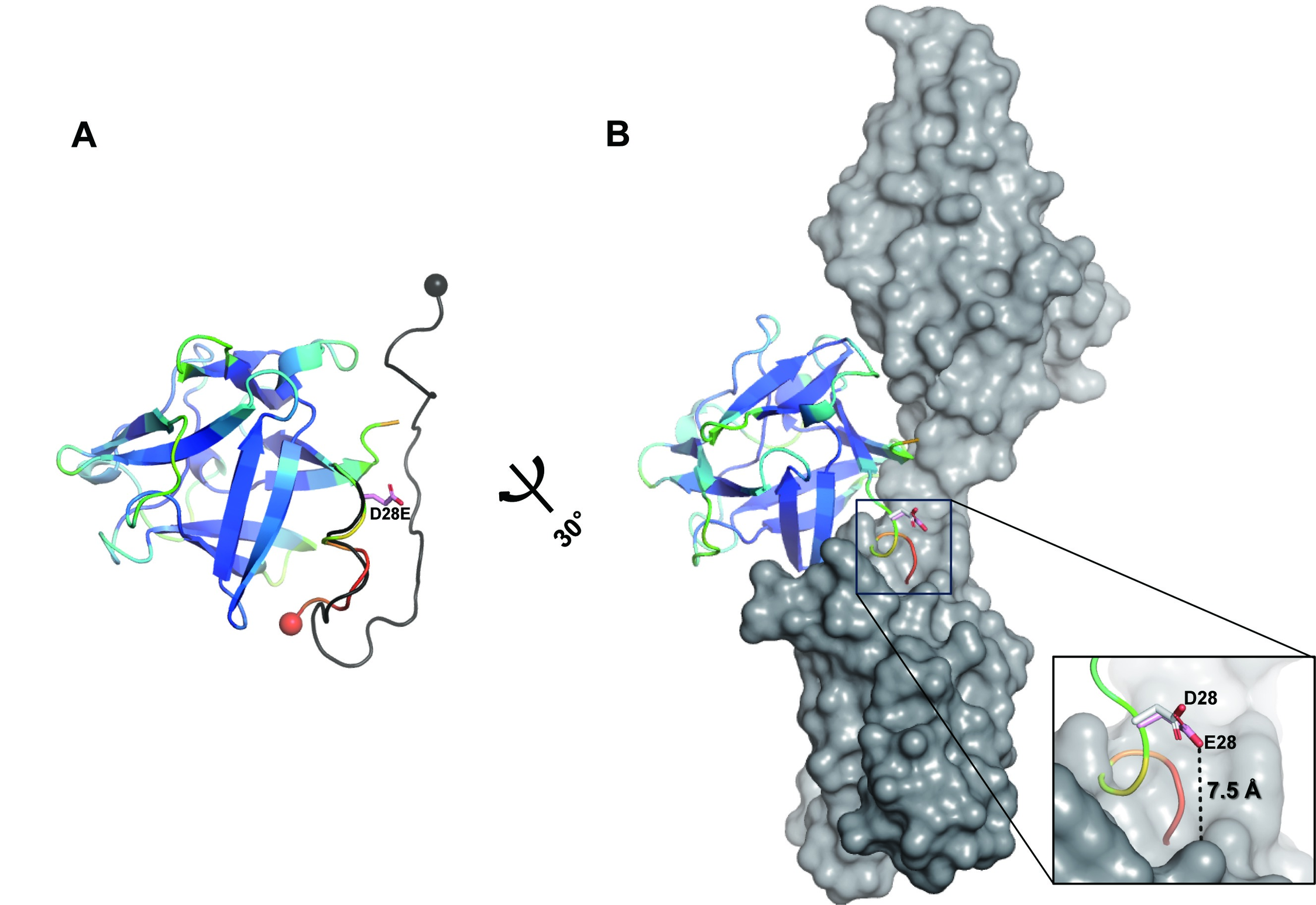

Supplement: S3 Fig — (A) Superimposition between the FGF2-M1 structure and the NMR structure (PDB code: 1BLA). Their N-terminus are shown as spheres. For clarity, only the N-terminal loop (residues 1–29) of the NMR structure is shown as black, while the full structure of FGF2-M1 is shown with B-factor representation. The color spectrum (red to blue) represents a range of B-factor values (43.63 to 9.02), which corresponds to a range of B’-factor values (3.38 to -1.28). The D28E mutation in FGF2-M1 is shown as a pink stick with a label. (B) Superposed model structure of the FGF2-M1/FGFR1c complex. From the template (PDB code: 1CVS), D28 is shown as a white stick with a label and FGFR1c is also depicted in a grey surface representation. The distance between the E28 side-chain and the surface of FGFR1c is indicated by a black dashed line. (TIF) [file pone.0307499.s008.tif]

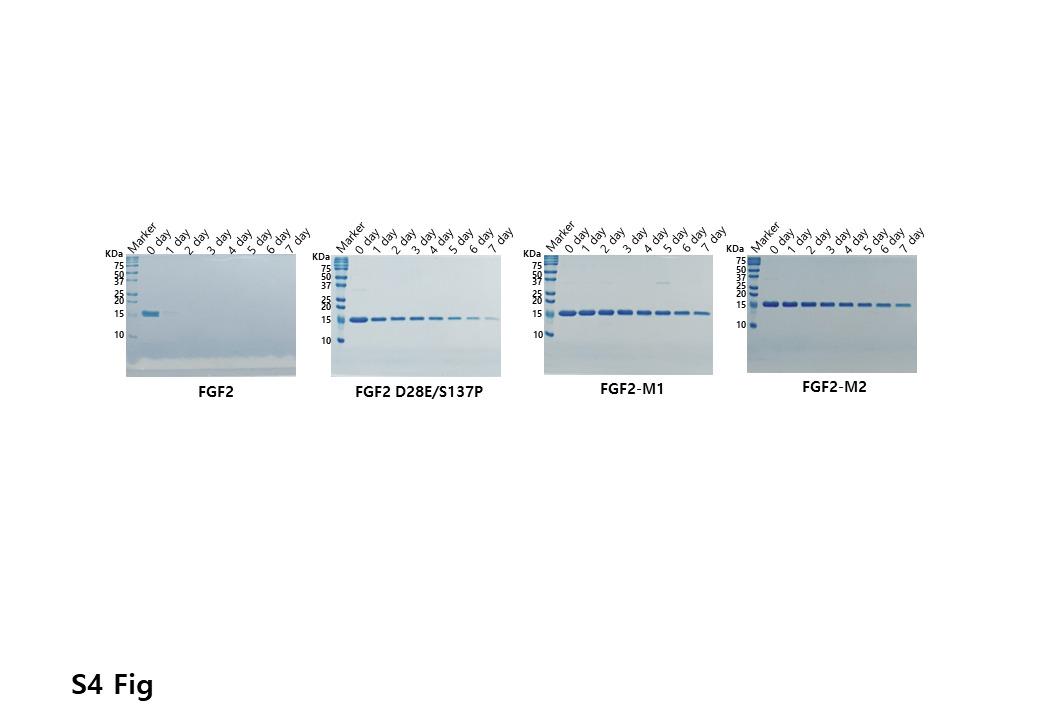

Supplement: S4 Fig — The FGF2 mutants were incubated at 45 °C for 7 days and checked for thermal stability using 15% SDS-PAGE. (TIF) [file pone.0307499.s009.tif]

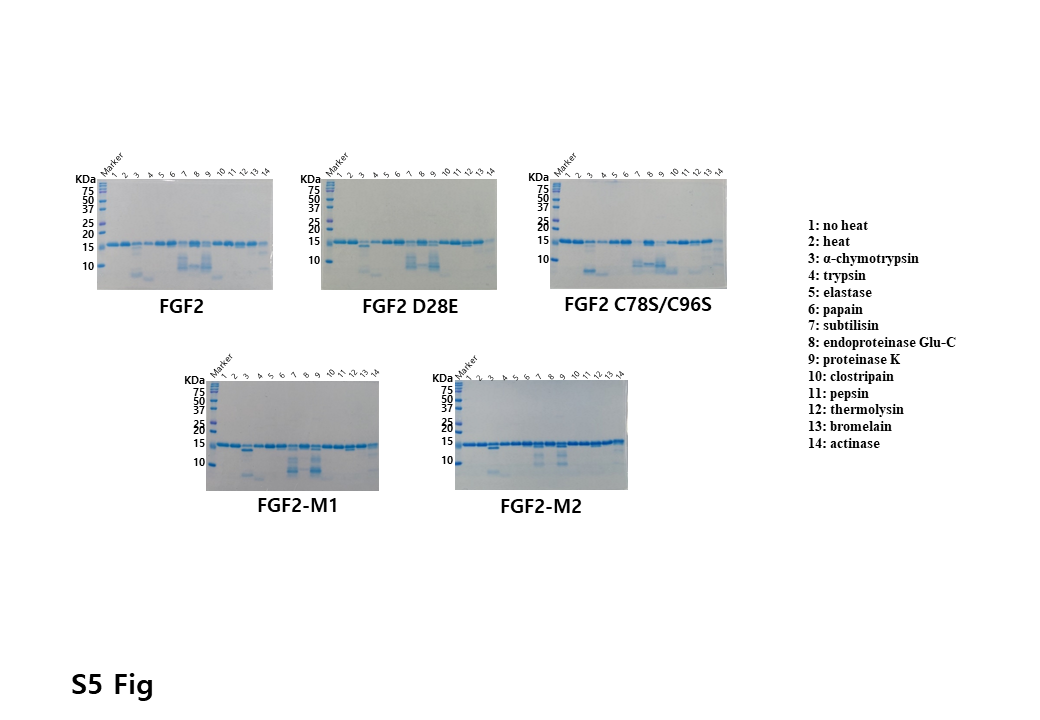

Supplement: S5 Fig — FGF2 variants (FGF2, FGF2 D28E, FGF2 C78S/C96S, FGF2-M1, FGF2-M2) were mixed with 12 different proteases and quantified using SDS-PAGE gel. Protease reactions were performed at 37 °C for 3 h. M: protein size marker, Lanes 1 and 2 represent unincubated and incubated protein samples, respectively. Lines 3: trypsin, 4: elastase, 5: chymotrypsin, 6: papain, 7: subtilisin, 8: endoproteinase Glu-C, 9: protease K, 10: clostripain (endoproteinase-Arg-C), 11: pepsin, 12: thermolysin, 13: bromelain, 14: actinase. (TIF) [file pone.0307499.s010.tif]

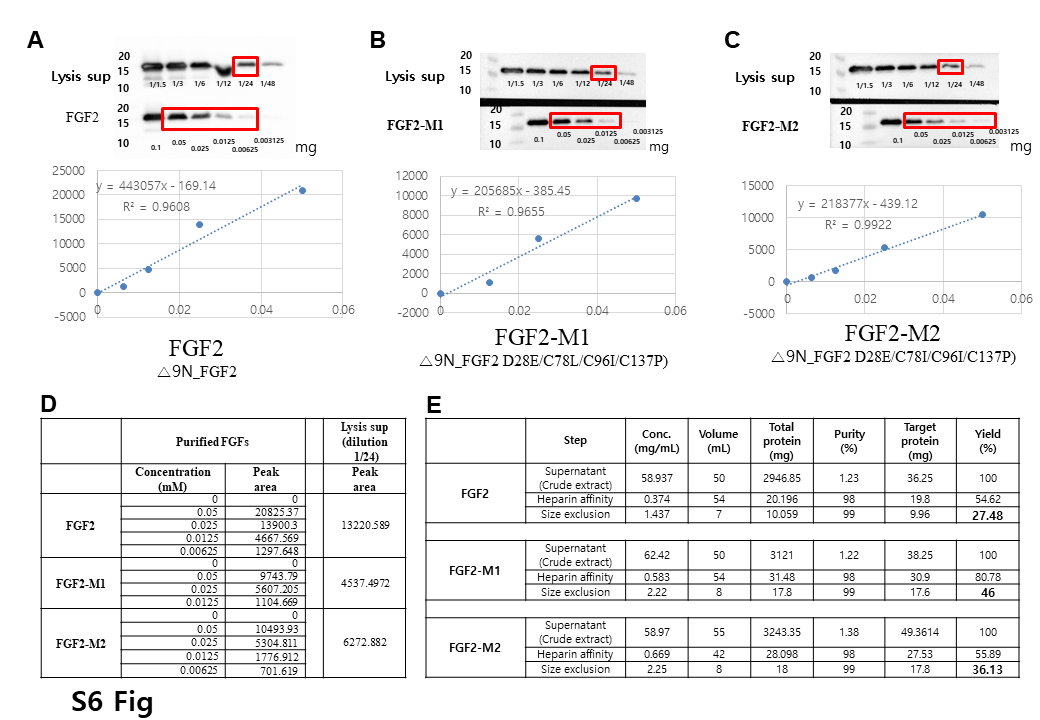

Supplement: S6 Fig — Calculation of (A) FGF2, (B) FGF2-M1, and (C) FGF2-M2 concentrations in supernatants of crude extracts by western blot. (D) Using the Western blot method, I calculated the standard curves for S6A-S6C Fig. To determine the peak levels of FGF2, I used the supernatant diluted 1/24 from the lysed cells. (E) production comparison table for each stage of FGF2s purified in two stages. (TIF) [file pone.0307499.s011.tif]
